# Deciphering Membrane‐Protein Interactions and High‐Throughput Antigen Identification with Cell Doublets

**DOI:** 10.1002/advs.202305750

**Published:** 2024-02-11

**Authors:** Yuqian Wang, Zhe Wang, Juan Yang, Xiaobo Lei, Yisu Liu, Luke Frankiw, Jianwei Wang, Guideng Li

**Affiliations:** ^1^ National Key Laboratory of Immunity and Inflammation Suzhou Institute of Systems Medicine Chinese Academy of Medical Sciences & Peking Union Medical College Suzhou Jiangsu 215123 China; ^2^ Key Laboratory of Synthetic Biology Regulatory Element Suzhou Institute of Systems Medicine Chinese Academy of Medical Sciences & Peking Union Medical College Suzhou Jiangsu 215123 China; ^3^ NHC Key Laboratory of Systems Biology of Pathogens Institute of Pathogen Biology Chinese Academy of Medical Sciences and Peking Union Medical College Beijing 100730 China; ^4^ Department of Pediatrics Boston Children's Hospital Boston MA 02115 USA

**Keywords:** doublets, membrane‐protein interaction, pMHC, T cell antigen discovery, TCR

## Abstract

Deciphering cellular interactions is essential to both understand the mechanisms underlying a broad range of human diseases, but also to manipulate therapies targeting these diseases. Here, the formation of cell doublets resulting from specific membrane ligand‐receptor interactions is discovered. Based on this phenomenon, the study developed DoubletSeeker, a novel high‐throughput method for the reliable identification of ligand‐receptor interactions. The study shows that DoubletSeeker can accurately identify T cell receptor (TCR)‐antigen interactions with high sensitivity and specificity. Notably, DoubletSeeker effectively captured paired TCR‐peptide major histocompatibility complex (pMHC) information during a highly complex library‐on‐library screening and successfully identified three mutant TCRs that specifically recognize the MART‐1 epitope. In turn, DoubletSeeker can act as an antigen discovery platform that allows for the development of novel immunotherapy targets, making it valuable for investigating fundamental tumor immunology.

## Graphical abstract

This study introduces DoubletSeeker, an innovative method to screen ligand‐receptor interactions by examining the formation of cell doublets from membrane protein interactions. DoubletSeeker demonstrates efficiency in identifying various ligand‐receptor interactions, such as TCRs and pMHCs, with high sensitivity and specificity. Notably, when coupled with single‐doublet sequencing technology, DoubletSeeker enables the decoding of paired TCR‐pMHC information in a library‐on‐library screening approach.

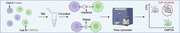

## 1 Introduction

Intercellular communication is essential for the cell‐fate decisions that drive both physiological and pathological processes in a wide range of fields, from tissue homeostasis to development to immunology.^[^ ^1^ ^]^ Thus, decoding the interactions that drive intercellular communication is extremely important to understand the underlying physiology and pathology, as well as to facilitate the design of potential drug targets. With respect to the latter, the complex interactions between peptide major histocompatibility complex (pMHC) ligands and T cell receptors (TCRs) are of particular interest due to the implications they have for the field of cancer immunotherapy. With the advance of high‐through microscopy and single‐cell‐based molecular analysis, a variety of methods have been developed that allow for the investigation of membrane protein‐protein interactions (PPIs), such as PIC‐seq,^[^ ^2^ ^]^ PUP‐IT,^[^ ^3^ ^]^ and BioID.^[^ ^4^ ^]^ However, it remains unclear whether these methods are sensitive enough to capture the complex interactions between pMHC ligands and TCRs.

TCR repertoire diversity, especially in the complementarity‐determining regions (CDR1, CDR2, and CDR3), is generated by a process of somatic recombination. Accurate interactions between these CDRs and the pMHC complex account for specific TCR‐pMHC recognition. The CDR1 and CDR2 loops interact mainly with the MHC molecules, while the hypervariable CDR3 loop directly contacts the antigen peptides.^[^ ^5^ ^]^ The complicated epitope recognition of TCRs^[^ ^6^ ^]^ and the enormous diversity of TCR repertoire in each individual^[^ ^7^ ^]^ demand methods that allow screening the TCR‐pMHC interactions in a high‐throughput manner. Thus, some high‐throughput approaches have been recently established for mapping TCR‐antigen interactions, including DNA‐barcoded pMHC tetramer,^[^ ^8^ ^]^ yeast display platform,^[^ ^9^ ^]^ cell‐based approaches^[^ ^10^ ^]^ (e.g., trogocytosis^[^ ^11^ ^]^) and engineered retrovirus‐based systems (ENTER and RAPTR).^[^ ^12^, ^13^ ^]^ These approaches enable the identification of TCRs that recognize given antigens or the antigen recognized by a given TCR. However, most of the current methods are still constrained by extensive antigenic peptide synthesis, poor sensitivity to low‐affinity antigens, inability to perform large‐scale “library‐on‐library” screening, and labor intensity.

Doublets (a pair of two cells) are routinely observed in flow cytometry or single‐cell analysis, but are often considered technological artifacts and are thus discarded, or ignored before gathering data.^[^ ^14^ ^]^ However, doublets are not simply technological artifacts of the flow cytometry process but rather represent accurate physical cell–cell contact.^[^ ^15^ ^]^ Due to physical cell–cell interactions, doublets were commonly observed in many different cell types, including T cells with dendritic cells (DCs) and tumor cells.^[^ ^16^ ^]^ The specificity of doublets formation between the T cells and antigen‐presenting cells (APCs) is likely dictated by the immunological synapses created via the TCRs' recognition of pMHCs.^[^ ^17^ ^]^ In the present study, we utilized the phenomenon of doublet formation to develop a new method, DoubletSeeker, to facilitate the library‐on‐library screening of the ligand‐receptor interactions. DoubletSeeker combines doublets formation and single‐doublet sequencing technology, allowing high‐throughput capture of ligand‐receptor pairing information. We showed that DoubletSeeker enables the characterization of a variety of membrane protein‐protein interactions, including interactions between cytokine receptors and membrane‐anchored ligands, immune costimulatory or immunosuppressive molecules and their respective ligands, antibodies, and antigens, as well as TCRs and pMHCs. We further verified the high sensitivity and specificity of DoubletSeeker in identifying T cells specific for a given antigen and in discovering antigens for a given TCR. Finally, we have successfully demonstrated the capability of DoubletSeeker to capture pairs of TCR‐pMHC through a comprehensive library‐on‐library screen.

## 2 Results

### 2.1 Establishing DoubletSeeker to Capture Membrane Protein–Protein Interactions

**Figure 1:**
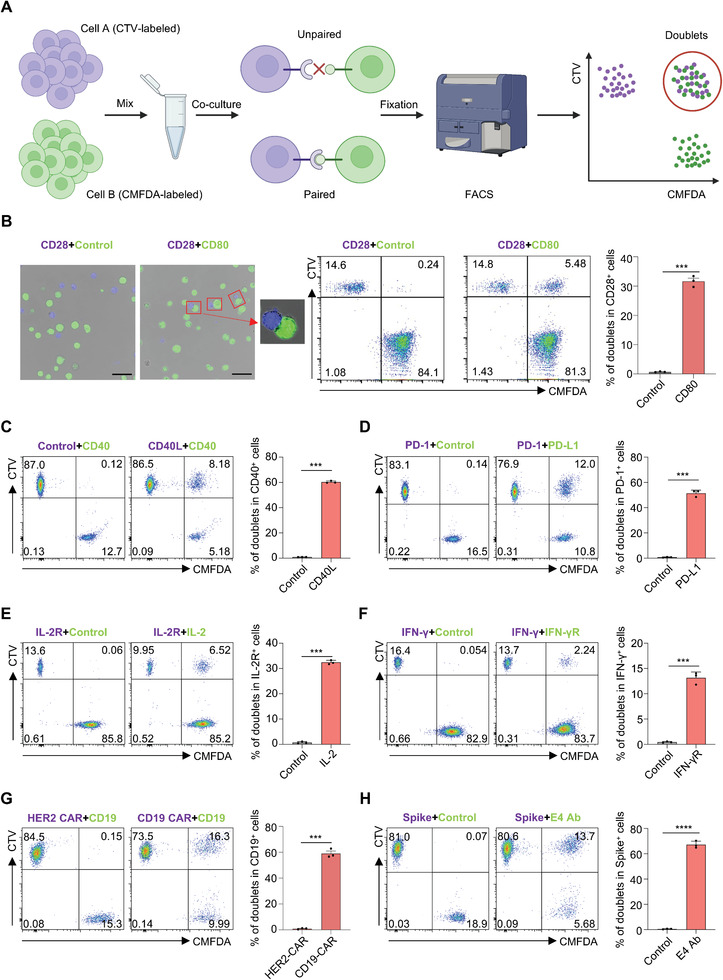
DoubletSeeker allows for the detection of a broad range of interactions between various ligand‐receptor types by specifically targeting cell doublets. A) Schema of the DoubletSeeker method. Cell A, expressing the ligand of interest, and cell B, expressing a receptor, were labeled with distinct fluorescence CellTrace dyes. Subsequently, the labeled cells were co‐incubated, and the interaction between the ligand and receptor on the cells led to the formation of cell doublets. In this diagram, cell A was labeled with CTV, while cell B was labeled with CMFDA. The FACS technique was utilized to capture the double fluorescent doublets. B) Detection of doublets formation by confocal microscopy (left) and by flow cytometry (right) after CD28‐Jurkat cells (CTV‐labeled, blue) co‐incubated with either control K562 or CD80‐K562 cells (CMFDA‐labeled, green). Red rectangle boxes mark the presence of doublets. Scale bars, 50 µm. Representative flow cytometry plots for doublet identification are presented in the middle. Bar plots presented to the right show the percentage of doublets for CD28‐Jurkat cells. (C to H) Formation of doublets was tested across a broad range of receptors and targeting ligands, including C) CD40/CD40L, D) PD1/PD‐L1, E) IL‐2R/IL‐2, F) IFN‐γR/IFN‐γ, G) CD19‐CAR/CD19, and the Spike protein of SARS‐CoV2 with its specific antibody E4‐Ab (H). Representative flow cytometry plots for doublet formation are presented on the left. Bar plots on the right show the percentage of doublets within the indicated ligand or receptor‐expressing cells. CTV: CellTrace Violet, CMFDA: CellTrace CMFDA. Data are represented as the means ± SEM. *n* = 3. ^**^ *p* < 0.01, ^***^ *p* < 0.001, and ^****^ *p* < 0.0001 (unpaired two‐tailed Student's t‐test). Data are representative of three independent experiments (B–H).

Here, we sought to develop a method, termed DoubletSeeker, that can simultaneously capture interacting cell pairs and uses sequencing technology to decode the information of both the ligand and receptor in these cell pairs (**Figure** **1**A). We first established cell lines stably expressing genes encoding either receptors or the respective ligand, including Jurkat cells expressing CD28 and K562 cells expressing CD80. To minimize the potential signal‐to‐noise ratio of the doublet, we compared the fluorescent intensity of fluorescent proteins and tracer dyes (CellTrace Violet‐CTV and CellTracke Green‐CMFDA) by flow cytometry. We found that the tracer dyes‐labeled cells are much brighter than the cells expressing fluorescent proteins and the tracer dyes do not leak from cells (Figure S1A,B, Supporting Information), making the tracer dyes suitable for long‐term cell labeling and doublet tracking. Indeed, tracer dye‐labeled cells displayed less non‐specific formation of doublets (Figure S1C,D, Supporting Information). To directly test the formation of cell doublets upon specific ligand‐receptor interaction, we co‐incubated tracer dyes‐labeled CD28‐Jurkat cells with control K562 cells or CD80‐K562 cells and examined the cell doublets (CTV^+^CMFDA^+^). Using both microscopy and flow cytometry, we observed significant amounts of cell doublets formed upon the incubation of CD28‐Jurkat cells with cognate CD80‐K562 cells, but not with control K562 cells (Figure 1B), suggesting cell doublets are sufficiently stable for flow cytometry tracing. We next tested if cell doublets could be formed among different ligand‐receptor interactions. We observed the formation of doublets through various ligand‐receptor interactions, including interactions between immune‐associated proteins and their receptors (CD40L/CD40 and programmed death 1(PD1)/programmed death ligand 1(PD‐L1) and CD28/CD86), glycosylphosphatidylinositol(GPI)‐modified membrane‐anchored cytokines and their receptors (interleukin‐2(IL‐2)/interleukin‐2 receptor(IL‐2R) and interferon‐γ(IFN‐γ)/interferon‐γ receptor(IFN‐γR)), viral protein and their receptors (the Spike protein of severe acute respiratory syndrome coronavirus 2(SARS‐CoV‐2) and its different receptors, angiotensin converting enzyme(ACE2),^[^ ^18^ ^]^ asialoglycoprotein receptor 1(ASGR1),^[^ ^19^ ^]^ neuropilin(NRP1),^[^ ^20^ ^]^ kringle containing transmembrane protein 1(KREMEN1),^[^ ^19^ ^]^ tyrosine‐protein kinase receptor(AXL),^[^ ^21^ ^]^ C‐type lectin domain family 10 member A(CLEC10A),^[^ ^19^ ^]^ dipeotidyl peptidase‐4(DPP4),^[^ ^22^ ^]^ low‐density lipoprotein receptor(LDLR),^[^ ^23^ ^]^ and so on^[^ ^24^ ^]^ (Figure 1C–F; Figure S2A–G, Supporting Information). In addition, the interaction of membrane proteins with antibodies can also be captured by doublet formation, including chimeric antigen receptor (CAR) and their target membrane proteins (CD19‐CAR/CD19, epidermal growth factor receptor(EGFR)‐CAR/EGFR, human epidermal growth factor receptor(HER2)‐CAR/HER2), and the Spike protein of SARS‐CoV‐2 and its neutralizing antibody (E4) (Figure 1G,H; Figure S2H, Supporting Information). Importantly, the introduction of recombinant E4 antibody into the coculture medium effectively blocks the formation of doublets between Spike protein and its receptor ACE2 (Figure S2I, Supporting Information). Thus, the DoubletSeeker method can be used to capture various membrane protein interactions.

### 2.2 Identification of Interactions between TCR‐pMHC by DoubletSeeker

**Figure 2:**
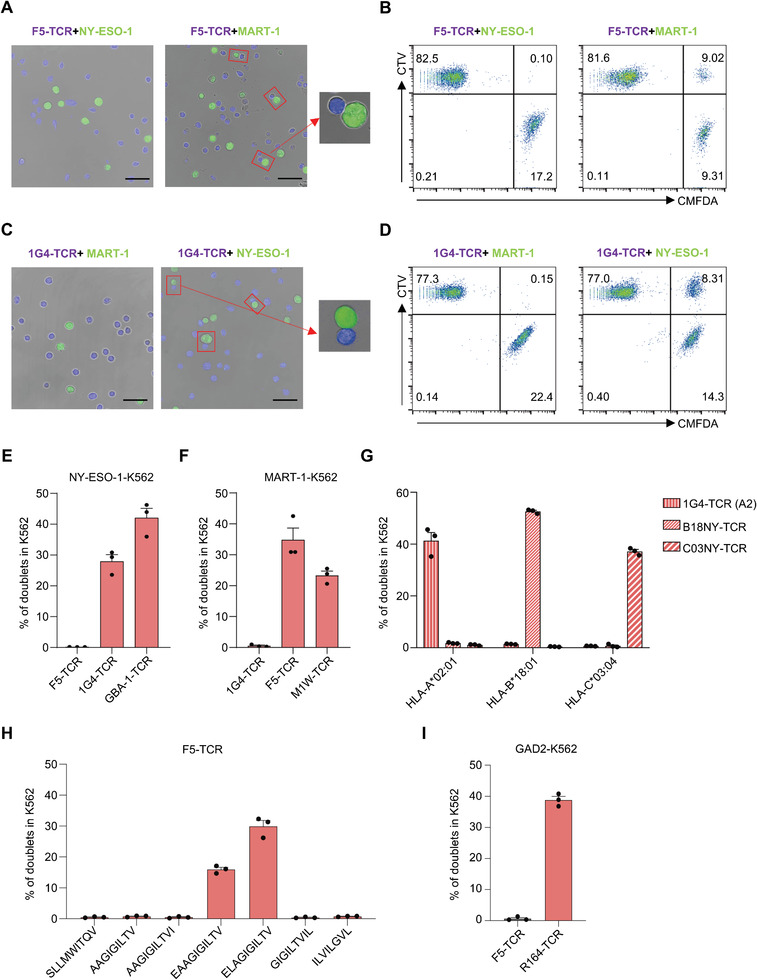
DoubletSeeker serves as a powerful tool for the identification of interactions between the TCRs and pMHCs. A–D) Doublets formed by co‐incubation of CTV‐labeled F5‐TCR‐Jurkat (A,B) or 1G4‐TCR‐Jurkat (C,D) cells with CMFDA‐labeled NY‐ESO‐1‐K562 or MART‐1‐K562 cells (TCR‐expressing cells: SCT‐expressing cells = 5:1) were analyzed by confocal microscopy (A,C) and flow cytometry (B,D). F5‐TCR is paired with MART‐1‐SCT, and 1G4‐TCR is paired with NY‐ESO‐1‐SCT. Red rectangle boxes mark the presence of doublets. Scale bars, 50 µm. E) Percentage of doublets in NY‐ESO‐1‐K562 cells after co‐incubation with their cognate 1G4‐TCR‐Jurkat, GBA‐1‐TCR‐Jurkat or noncognate F5‐TCR‐Jurkat cells (TCR‐expressing cells: SCT‐expressing cells = 5:1) was analyzed by flow cytometry. F) Percentage of doublets in MART‐1‐K562 cells, following co‐incubation with Jurkat cells expressing the cognate F5‐TCR or M1W‐TCR (TCR‐expressing cells: SCT‐expressing cells = 5:1), was analyzed by flow cytometry. G) Antigen‐specific doublet formation between K562 expressing HLA‐A*02:01, HLA‐B*18:01 or HLA‐C*03:04 restricted NY‐ESO‐1 epitopes and Jurkat cells expressing their cognate TCRs (TCR‐expressing cells: SCT‐expressing cells = 5:1). H) Comparison of doublets formation in K562 cells expressing diverse HLA‐A*02:01‐restricted MART‐1 peptide variants after co‐incubation with F5‐TCR‐Jurkat cells. I) Percentage of doublets in K562 cells expressing MHC‐II restricted GAD2 antigen after co‐incubation with cognate R164‐TCR‐Jurkat or noncognate F5‐TCR‐Jurkat cells (TCR‐expressing cells: SCT‐expressing cells = 5:1) was analyzed by flow cytometry. CTV: CellTrace Violet, CMFDA: CellTrace CMFDA. Data are represented as the means ± SEM. *N* = 3. Data are representative of three independent experiments (A–I).

The binding strength between TCR and pMHC is generally very low.^[^ ^25^ ^]^ Thus, we examined if the interaction of TCR and pMHC would be sufficiently stable for FACS tracing. By co‐incubation of Jurkat cells expressing F5‐TCR with K562 cells expressing its cognate peptide HLA‐A*02:01/MART‐1_26‐35(A27L)_, or K562 cells expressing HLA‐A*02:01/NY‐ESO‐1_157‐165(C165V)_ as single‐chain trimers (SCTs),^[^ ^26^ ^]^ we observed antigen‐specific forming of cell doublets of F5‐TCR‐Jurkat cells with MART‐1‐K562 cells but not with NY‐ESO‐1‐K562 cells (**Figure** **2**A). Notably, these TCR‐pMHC interacting cell doublets were very stable and can be acquired by flow cytometry (Figure 2B). Similarly, the co‐incubation of 1G4‐TCR‐Jurkat cells with cognate NY‐ESO‐1‐K562 cells also led to the stable and specific formation of cell doublets (Figure 2C,D). Coreceptor CD8 acts to stabilize the binding of TCR to the pMHC complex.^[^ ^27^ ^]^ Indeed, we observed significantly decreased percentages of cell doublets when CD8 was not expressed in F5‐TCR‐Jurkats or 1G4‐TCR‐Jurkat cells (Figure S3A,B, Supporting Information).

Next, we examined the formation of doublets in SCT‐K562 cells upon co‐incubation with TCR‐Jurkat cells at different ratios or for different time‐periods. We observed a gradual increase in the percentage of cell doublets in MART‐1‐ or NY‐ESO‐1‐K562 cells as the proportions of their cognate F5‐ or 1G4‐TCR‐Jurkat cells increased (Figure S3C,D, Supporting Information). Furthermore, after 30 min of co‐incubation, approximately 40–50% of MART‐1‐K562 and NY‐ESO‐1‐K562 cells formed doublets with their cognate TCR‐Jurkat cells. However, with extended co‐incubation beyond 30 min, the formation of doublets gradually decreased (Figure S3E,F, Supporting Information). In addition, the percentage of nonspecific doublets remained ≈1% and showed minimal changes across different conditions, further demonstrating that the formation of doublets was TCR‐pMHC interaction specific.

In addition to F5‐ and 1G4‐TCR, we also observed specific doublets formation for five additional TCR‐pMHC cognate pairings, including a novel A2‐restricted NY‐ESO‐1‐specific TCR (GBA‐1‐TCR), a low‐affinity A2‐restricted MART‐1‐specific TCR (M1W‐TCR), and two HLA‐B*18:01‐ and C*03:04‐restricted NY‐ESO‐1‐specific TCRs (Figure 2E–G). Of note, even low‐affinity M1W‐TCR displayed a substantial percentage of cell doublets, suggesting the DoubletSeeker method would be able to enrich lower affinity TCR‐pMHC interactions. We next evaluated the sensitivity of DoubletSeeker in differentiating the recognition of peptide variants by a TCR. By incubating F5‐TCR‐Jurkat cells with A2‐K562 cells that express different MART‐1 peptide variants at the same level, we found that there were more cell doublets formed in the co‐incubation of F5‐TCR‐Jurkat cells with K562 cells presenting heteroclitic MART‐1 peptide (ELAGIGILTV) than that with K562 cells presenting native low avidity peptide (EAAGIGILTV), suggesting the extent of doublets formation correlates with the avidity of the TCR–pMHC interaction (Figure 2H). Of note, such correlation is absent in receptor‐ligand interactions and antibody‐antigen interactions (Figure S3G, Supporting Information). By co‐culturing K562‐ACE2 cells and Jurkat‐Spike cells with varying levels of Spike protein expression, we observed a substantial increase in the percentage of cell doublet formation when target protein was expressed at higher levels on the cells, revealing an association between doublets formation and protein expression levels (Figure S3H,I, Supporting Information). Therefore, we hypothesize that optimizing TCR or peptide presentation levels could enhance the applicability of DoubletSeeker in primary cells. To investigate the impact of increased antigen presentation on doublet formation, we treated B16F10 and B16F10‐OVA tumor cells with IFN‐γ, which is known to upregulate MHC class I expression and antigen presentation. We observed a significant increase in doublet formation (14.3%) during coculture with OVA_SIINFEKL_‐specific primary OT‐I T cells, in comparison to untreated B16F10‐OVA cells (4.02%) and control B16F10 cells (1.15%). Additionally, we found that ≈50% of OVA_SIINFEKL_‐SCT overexpressing B16F10 (B16F10‐OVA SCT) cells formed doublets with primary OT‐I T cells, further supporting the notion that high levels of surface antigen expression increase doublet formation (Figure S3J,K, Supporting Information). In addition to MHC‐I restricted TCRs, we also tested the ability of DoubletSeeker to detect MHC‐II restricted TCR‐antigen interactions. We found that Jurkat cells expressing MHC‐II restricted R164‐TCR can also form stable doublets with K562 cells presenting the cognate GAD2 peptide (Figure 2I). These findings demonstrate that DoubletSeeker is capable of capturing specific interactions between TCRs and pMHCs.

### 2.3 Specificity and Sensitivity of DoubletSeeker for Resolving Cognate Antigens

**Figure 3:**
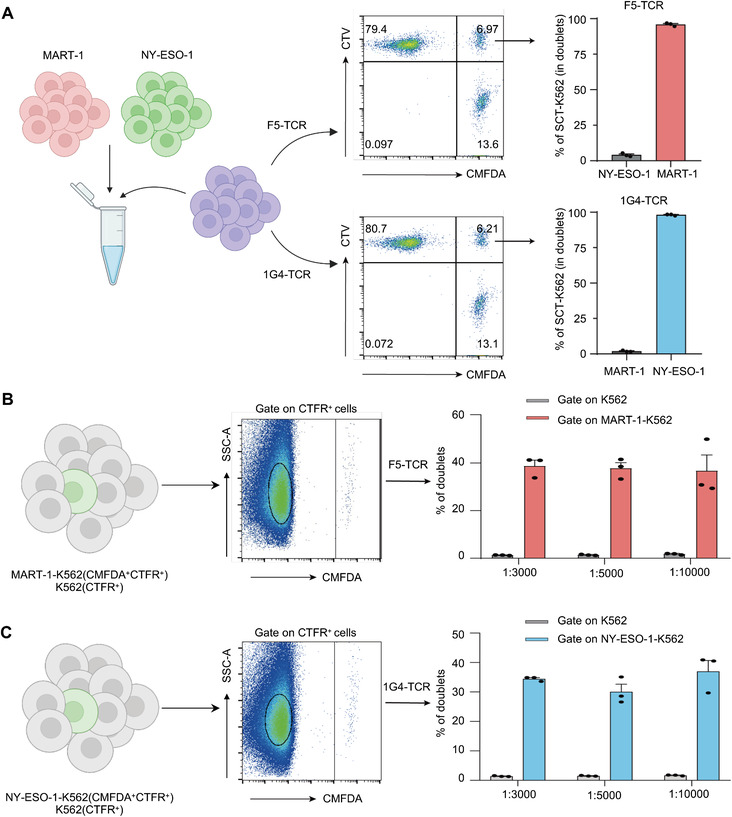
DoubletSeeker demonstrates remarkable specificity and sensitivity in identifying cognate antigens for a given TCR. A) Schematic and representative flow cytometry plots for a 1:1 mixture of MART‐1‐K562 and NY‐ESO‐1‐K562 cells (CMFDA‐labeled) co‐incubated with either CTV‐labeled F5‐TCR‐Jurkat cells (top) or 1G4‐TCR‐Jurkat cells (bottom) (TCR‐expressing cells: SCT‐expressing cells = 5:1). K562 cells expressing the cognate antigens were additionally labeled with CTFR. The percentage quantification of each SCT‐K562 cell in the doublet population is presented on the right. B,C) Schematic and representative flow cytometry plots for the mixture containing cognate B) MART‐1‐K562 or C) NY‐ESO‐1‐K562 cells with noncognate K562 cells at different ratios (1:3000, 1:5000, 1:10 000) co‐incubated with either F5‐TCR‐Jurkat cells (B) or 1G4‐TCR‐Jurkat cells (C) (TCR‐expressing cells: SCT‐expressing cells = 1:1). The percentage quantification of doublets in each K562 cells are presented on the right. MART‐1‐K562 and NY‐ESO‐1‐K562 cells were labeled with CMFDA and CTFR, whereas K562 cells were labeled with CTFR. CTV: CellTrace Violet, CMFDA: CellTrace CMFDA, CTFR: CellTrace Far‐red. Data are represented as the means ± SEM. *n* = 3. Data are representative of three independent experiments.

We next assessed DoubletSeeker's ability to distinguish antigen‐expressing target cells from cells expressing noncognate antigens for a given TCR. We co‐incubated CMFDA‐labeled NY‐ESO‐1‐K562 cells and MART‐1‐K562 cells (in which target cells were additionally labeled with CellTrace Far‐red‐CTFR) in a 1:1 ratio with CTV‐labeled Jurkat cells expressing F5‐ or 1G4‐TCR respectively. Target MART‐1‐K562 cells for F5‐TCR were substantially enriched (98%) in the cell doublets when the NY‐ESO‐1‐ and MART‐1‐K562 cell mixture was co‐incubated with F5‐TCR‐Jurkat cells (**Figure** **3**A, upper panel). Similarly, the enrichment of NY‐ESO‐1‐K562 cells was observed when the mixture was incubated with 1G4‐Jurkat cells (Figure 3A, lower panel). Thus, DoubletSeeker can specifically enrich cognate TCR–pMHC cell pairs, allowing the efficient identification of on‐target cells from noncognate epitope‐presenting cells. To further validate the sensitivity of DoubletSeeker in capturing cognate target cells, we conducted a proof‐of‐concept library screening. We mixed on‐target MART‐1‐K562 (CTFR^+^CMFDA^+^) and control K562 cells (CTFR^+^CMFDA^−^) at different ratios, and then cocultured this mixture with CTV‐labeled F5‐TCR‐Jurkat cells specific to MART‐1. We then quantified the percentage of cell doublets formed by MART‐1‐K562 and control K562 cells with F5‐TCR‐Jurkat cells (CTFR^+^CMFDA^+^CTV^+^ versus CTFR^+^CMFDA^−^CTV^+^). A similar experiment was also performed using NY‐ESO‐1‐K562 cells and control K562 cells in combination with 1G4‐TCR‐Jurkat cells. We found that, compared with control K562 cells, a remarkably higher percentage of target antigen‐presenting K562 cells (≈40%) formed doublets with their cognate TCR‐Jurkat cells, even when the target cells were at a ratio as low as 1:10 000 (Figure 3B,C; Figure S4, Supporting Information). In addition, DoubletSeeker also effectively isolates various interacting cells, including CD28/CD80, CD28/CD86, CD19‐CAR/CD19, and CD40/CD40L interactions (Figure S5, Supporting Information). These results demonstrated the high specificity and sensitivity of DoubletSeeker for resolving cognate antigens for a given TCR, as well as a broad range of membrane protein interactions.

### 2.4 Identification of Cognate TCR Antigens by DoubletSeeker

**Figure 4:**
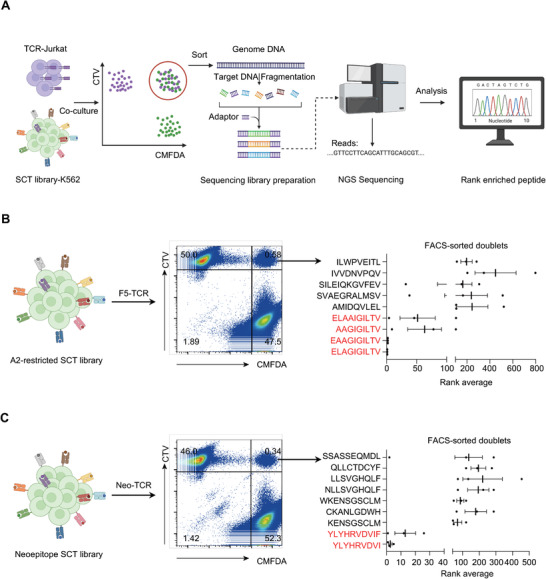
DoubletSeeker excels in identifying the cognate antigens for public F5‐TCR and neoantigen‐specific TCR. A) Schematic diagram of DoubletSeeker for screening TCR cognate epitopes from an SCT library. CTV‐labelled TCR‐expressing Jurkat cells co‐incubated with CMFDA‐labelled SCT library‐expressing K562 cells at a 1:1 ratio. Subsequently, FACS‐sorted (CTV^+^CMFDA^+^) cell doublets were then subjected to NGS to identify the enriched epitopes based on read counts. B,C) Schematic and representative flow cytometry plots for the identification of doublets in K562 cells expressing an A2‐restricted SCT library containing 12055 epitopes (B) and a neoepitope SCT library containing 3251 unique neoepitopes C) co‐incubated with F5‐TCR‐Jurkat (B) or Neo‐TCR‐Jurkat (C) cells (TCR‐expressing cells: SCT‐expressing cells = 1:1), respectively. Identification of enriched epitopes after one round of selection by NGS is shown on the right. The rank average is determined by calculating the ranking of each peptide based on its abundance among all peptides, from three independent experiments. The SCT library‐K562 cells were labeled with CMFDA, and the TCR‐Jurkat cells were labeled with CTV. Data are represented as the means ± SEM. *n* = 3.

With the key characteristics of DoubletSeeker validated, we next asked whether DoubletSeeker can be used to screen the candidate antigen for a given TCR in a more extensive library. We designed a strategy to utilize DoubletSeeker for the acquisition of FACS‐sorted cell doublets, which were then subjected to Next‐generation sequencing (NGS) analysis for the identification of target epitopes (**Figure** **4**A). We incubated CTV‐labeled F5‐TCR‐Jurkat cells with CMFDA‐labeled K562 cells expressing an A2‐restricted SCT cDNA library consisting of all known HLA‐A*02:01‐restricted epitopes, from the Immune Epitope Database (IEDB). FACS‐sorted (CTV^+^CMFDA^+^) cell doublets were then subjected to NGS to identify the enriched epitopes. After one round of doublet selection, the top four enriched epitopes that share similar sequences were known cognate ligands for the F5‐TCR (Figure 4B; Figure S6A, Supporting Information). These data demonstrate that DoubletSeeker can enrich cognate antigens and decode the cross‐reactive ligands with a similar sequence for a given TCR. We further tested the versatility of DoubletSeeker for neoantigen discovery using a neoantigen‐specific tumor‐reactivity Neo‐TCR that was isolated from a subject with metastatic melanoma. We used the Neo‐TCR as a surrogate for a tumor‐reactive orphan TCR and conducted co‐incubation experiments with CTV‐labeled Jurkat cells expressing Neo‐TCR and CMFDA‐labeled K562 cells transduced with a neoepitope SCT cDNA library containing 3251 predicted A2‐restricted neoepitopes (8 to 12 amino acids in length) corresponding to nonsynonymous mutations found in the tumor. Subsequent NGS sequencing and the ranking result of enriched epitopes showed that the top two hits were epitopes derived from USP‐7 with a nonsynonymous D798Y mutation (YLYHRVDVI and YLYHRVDVIF) (Figure 4C; Figure S6B, Supporting Information). These results demonstrate that the DoubletSeeker method is an efficient and effective method for the deorphanization of TCRs.

### 2.5 Identification of Antigen‐Specific TCRs by DoubletSeeker

**Figure 5:**
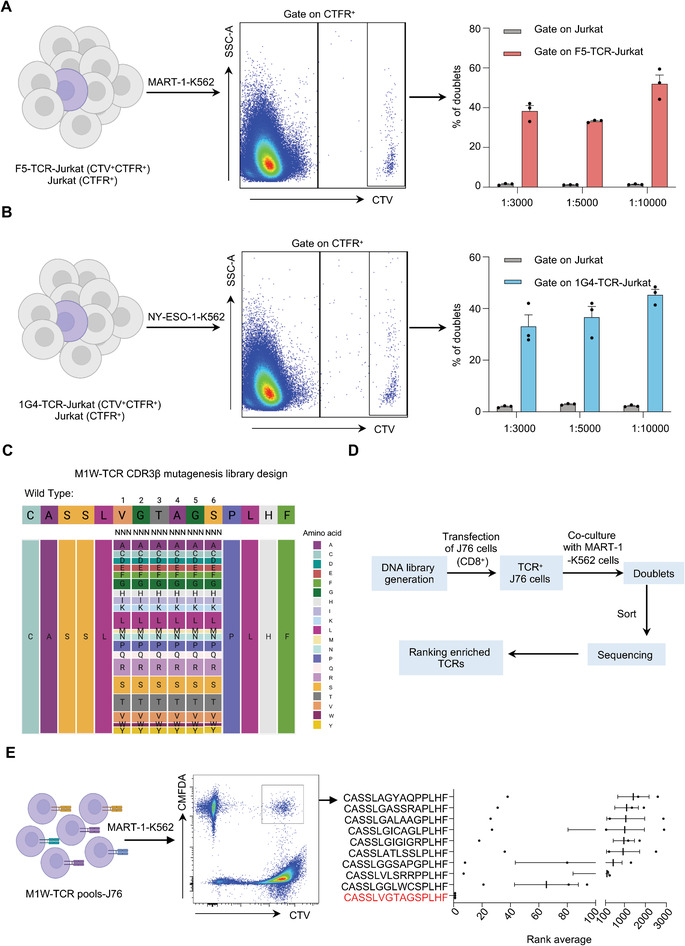
DoubletSeeker enables to identification of cognate TCRs for a given antigen. A,B) Schematic and representative flow cytometry plots for the mixture containing cognate A) F5‐TCR‐Jurkat or B) 1G4‐TCR‐Jurkat cells with control Jurkat cells at different ratios (1:3000, 1:5000, 1:10 000) co‐incubated with either A) MART‐1‐K562 or B) NY‐ESO‐1‐K562 cells labeled with CMFDA (TCR‐expressing cells: SCT‐expressing cells = 1:1). Bar plots that show the population of doublets in each Jurkat cell population are presented on the right. F5‐TCR‐Jurkat and 1G4‐TCR‐Jurkat cells were labeled with CTV and CTFR, whereas Jurkat cells were labeled with CTFR. C) Schematic for the design of the M1W‐TCR CDR3β combinatorial mutagenesis library. D) Strategy for screening MART‐1‐specific TCRs from the M1W‐TCR pools‐J76 library using DoubletSeeker. E) Schematic and representative flow cytometry plots for the identification of doublets in CD8‐J76 cells expressing the M1W‐TCR and its corresponding mutation library (pre‐mixed 0.1% WT M1W‐TCR virus) co‐incubated with MART‐1‐K562 cells (TCR‐expressing cells: SCT‐expressing cells = 1:1). Identification of enriched epitopes after one round of selection by NGS is shown on the right. The rank average is determined by calculating the ranking of each peptide based on its abundance among all peptides, from three independent experiments. The M1W‐TCR pools‐J76 library was labeled with CTV, and the MART‐1‐K562 cells were labeled with CMFDA. CTV: CellTrace Violet, CMFDA: CellTrace CMFDA, CTFR: CellTrace Far‐red. Data are represented as the means ± SEM. *n* = 3. Data are representative of three independent experiments (A,B,E).

Next, we explored if the DoubletSeeker method could enable the capture of TCRs that specifically recognize a given antigen. When CTV‐labeled F5‐TCR‐Jurkat cells and 1G4‐TCR‐Jurkat cells were co‐incubated with CMFDA‐labeled MART‐1‐ or NY‐ESO‐1‐K562 cells, significant formation of cell doublets was observed in the presence of their respective target antigens. Conversely, no such interaction was observed when co‐incubating the TCR‐Jurkat cells with K562 cells expressing noncognate antigens (Figure S7A,B, Supporting Information). This data indicates a specific interaction between TCR‐expressing Jurkat cells and K562 cells expressing their cognate antigens. To further assess the sensitivity of DoubletSeeker to capture antigen‐specific TCRs, we mixed F5‐ or 1G4‐TCR‐Jurkat (CTV^+^CTFR^+^) cells with control Jurkat cells (CTV^−^CTFR^+^) at different ratios and then co‐incubated them with MART‐1‐K562 cells and NY‐ESO‐1‐K562 cells (CMFDA^+^), respectively. We observed a significantly higher proportion of cell doublets (CTV^+^CTFR^+^CMFDA^+^) formed by F5‐TCR‐Jurkat and 1G4‐TCR‐Jurkat cells (≈40%) with targeted antigen‐presenting K562 cells compared to their control Jurkat counterparts, even when the ratio of antigen‐specific Jurkat cells was as low as 1:10 000 (**Figure** **5**A,B; and Figure S7C, Supporting Information).

We further generated a comprehensive mutant M1W‐TCR library by performing saturation mutagenesis on the six amino acids (AA) in the CDR3β region of M1W TCRβ, which pairs with the identical α chain of the M1W‐TCR (Figure 5C). The M1W‐TCR with its corresponding mutation library (mixed with 0.1% WT M1W‐TCR virus) was transduced into CD8‐expressed J76 cells (referred to as M1W‐TCR pools‐J76), an endogenous TCRα and TCRβ deficient Jurkat cell, at a multiplicity of infection (MOI) of 4 to achieve a high infection rate (Figure S7D, Supporting Information). Subsequently, TCR‐positive J76 cells were isolated and co‐incubated with MART‐1‐K562 cells, followed by FACS isolation of doublets and sequencing to identify the target TCRs. The top TCR enriched in the doublets was the wild‐type (WT) M1W‐TCR that recognizes the MART‐1_ELAGIGILTV_ epitope (Figure 5D,E). The other top enriched mutant TCRs exhibited much fewer reads in the doublets sequencing data and further validation experiments confirmed that none of these TCRs show strong interaction with the MART‐1_ELAGIGILTV_ epitope (Figure S8, Supporting Information). These results indicate that DoubletSeeker is capable of enriching antigen‐specific TCRs with high specificity and sensitivity.

### 2.6 The Library‐on‐Library Screening Capability of DoubletSeeker

To evaluate the library‐on‐library screening capability of DoubletSeeker, we mixed the TCR‐expressing Jurkat cells (F5‐TCR, 1G4‐TCR and Neo‐TCR) with their targeted SCT‐expressing K562 cells (MART‐1, NY‐ESO‐1 and USP‐7) at a 1:1 ratio. Each population of TCR‐Jurkat cells and SCT‐K562 cells were labeled with distinct fluorescent markers (Figure S9A, Supporting Information). We found a significant increase in the formation of cell doublets was observed in the paired TCR‐SCT expressing cells compared to the unpaired cells (Figure S9B, Supporting Information). This finding indicates the potential of DoubletSeeker in facilitating the identification of TCR‐pMHC interactions within complex cellular mixtures.

**Figure 6:**
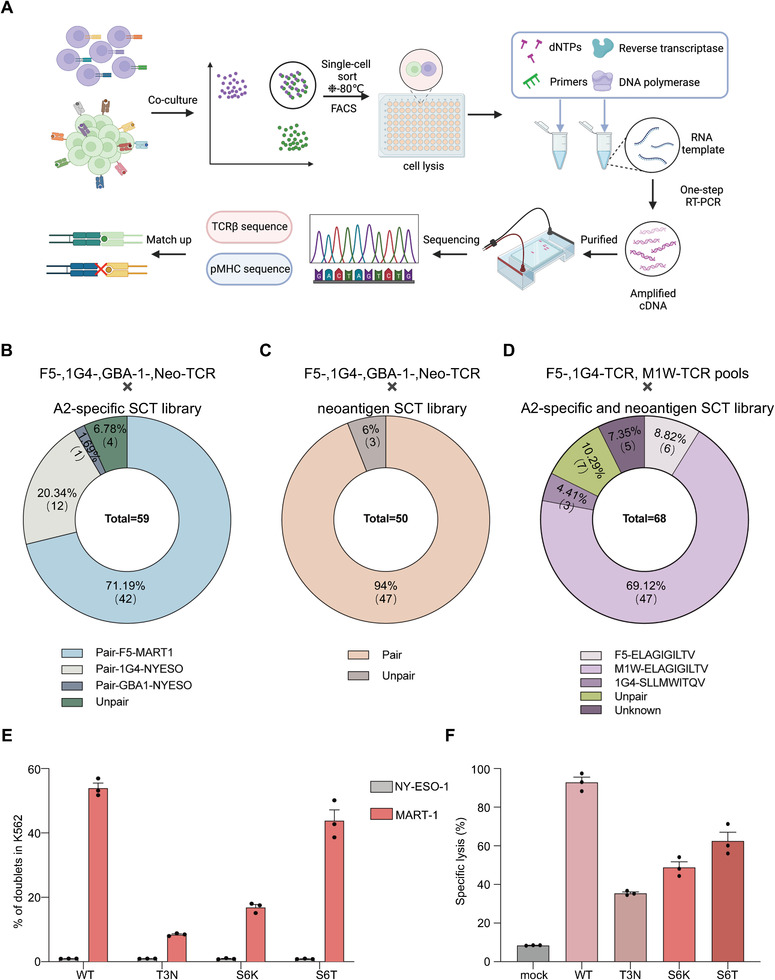
The library‐on‐library screening capability of DoubletSeeker. A) Schematic of DoubletSeeker for library‐on‐library screening of TCR‐pMHC interactions. Single doublets are sorted into individual wells of 96‐well plates, which are preloaded with cell lysis buffer, followed by freezing to lyse the doublet cells. Subsequently, the TCR and SCT information for each doublet is obtained through single‐cell PCR sequencing. B,C) The proportion of TCR‐pMHC pairs within the enriched doublets isolated from the co‐incubation of A2‐restricted SCT‐K562 library (B) or neoepitope SCT‐K562 library (C) with a mixture of F5‐, 1G4‐, GBA‐1‐ and Neo‐TCR‐Jurkat cells (TCR‐expressing cells: SCT‐expressing cells = 1:1). D) Proportion of TCR‐pMHC pairs within the enriched doublets isolated from the co‐incubation of the combined library of A2‐restricted SCT‐K562 and neoepitope SCT‐K562 with M1W‐TCR pools‐J76 cells, in the presence of a small proportion of F5‐ and 1G4‐TCR‐Jurkat cells (TCR‐expressing cells: SCT‐expressing cells = 1:1). E) Interaction between MART‐1‐K562 or NY‐ESO‐1‐K562 cells and J76 cells expressing M1W_WT_‐, M1W_T3N_‐, M1W_S6K_‐, or M1W_S6T_‐TCR as detected by DoubletSeeker. F) Cytotoxicity of T cells expressing M1W_WT_‐, M1W_T3N_‐, M1W_S6K_‐, or M1W_S6T_‐TCR against A2‐expressing K562 cells pulsed with MART‐1 peptide (ELAGIGILTV). The data are presented as percentage‐specific lysis rates. Data are represented as the means ± SEM. *n* = 3 (E and F).

Next, we devised a single doublet‐capturing strategy to validate the accuracy of DoubletSeeker in library‐on‐library screening, as illustrated in **Figure** **6**A. The A2‐restricted SCT of the K562 library, as mentioned in Figure 4B, was co‐incubated with a mixture of F5‐, 1G4‐, GBA‐1‐, and Neo‐TCR‐Jurkat cells (a mixture of 4 TCR‐Jurkat cells). After isolating single doublet using FACS, we conducted single‐cell PCR sequencing to obtain the TCR and antigen information for each doublet. Remarkably, the analysis of these sequenced doublets revealed that 93% of doublets containing well‐described TCR‐pMHC cognate pairings, including F5‐TCR/MART‐1‐SCT (71.19%), 1G4‐TCR/NY‐ESO‐1‐SCT (20.34%) and GBA‐1‐TCR/NY‐ESO‐1‐SCT (1.69%) (Figure 6B). Similarly, in the screening of the neoepitope SCT library with the 4 TCR‐Jurkat cell mixture, the Neo‐TCR/USP7‐SCT cognate pairings were predominantly enriched, accounting for over 94% of the isolated doublets (Figure 6C).

To further evaluate the efficacy of DoubletSeeker in enriching TCR‐pMHC interactions within a highly complex cellular mixture, we mixed the A2‐restricted antigen library expressing K562 cells and the neoepitope library expressing K562 cells together. This combined antigen library was subsequently co‐incubated with the low MOI transduced M1W‐TCR pools‐J76 cells, along with a small proportion of F5‐ and 1G4‐TCR‐Jurkat cells (Figure S9C, Supporting Information). After one round of doublets selection, our single‐doublets sequencing data revealed that DoubletSeeker successfully enriched the well‐described TCR‐pMHC pairings, including F5‐TCR/MART‐1‐SCT (8.82%), 1G4‐TCR/NY‐ESO‐1‐SCT (4.41%), and WT M1W‐TCR/MART‐1‐SCT (69.12%) cognate TCR‐pMHC interactions. In addition, we observed three mutant M1W‐TCRs (T3N, S6K and S6T) were also enriched with MART‐1_ELAGIGILTV_ epitope, categorized as unknown TCR‐pMHC pairings (Figure 6D). To verify the interaction of MART‐1_ELAGIGILTV_ epitope with the three mutant M1W‐TCRs, DoubletSeeker was performed to assess the formation of doublets by co‐incubating MART‐1‐K562 cells with J76 cells expressing M1W_T3N_‐, M1W_S6K_‐, or M1W_S6T_‐TCR, respectively. As expected, there was an increased percentage of doublets in MART‐1‐K562 cells co‐incubated with M1W_T3N_‐, M1W_S6K_‐, M1W_S6T_‐TCR‐Jurkat cells compared to noncognate NY‐ESO‐1‐K562 cells, despite the difference in TCR expression (Figure 6E; Figure S9D, Supporting Information). Notably, the three mutated TCR cells showed a reduced level of doublet formation, suggesting that the three M1W mutant TCRs have a lower binding affinity to MART‐1_ELAGIGILTV_. This was further validated by measuring the ability of the three mutant M1W‐TCRs to induce human T cell cytotoxicity upon recognition of A2‐expressing K562 cells pulsed with MART‐1 peptide (ELAGIGILTV) (Figure 6F). Therefore, DoubletSeeker demonstrates the high efficiency in capturing the cognate TCR‐pMHC pairs in a highly complex library‐on‐library screening.

## 3 Discussion

Cell‐to‐cell communications orchestrate complex biological processes like organismal development, tissue homeostasis, and immune responses. These communications rely on the interactions between ligands and their cell surface receptors. Deciphering these interactions is important for both fundamental research and drug discovery efforts. In this study, we developed DoubletSeeker, a novel and high‐throughput approach for identifying ligand‐receptor interactions found between various cell surface proteins such as cytokine receptors, immune costimulatory molecules, viral proteins (i.e., the spike protein of SARS‐CoV‐2), antibodies, and TCRs. Through a series of proof‐of‐concept experiments and screening, we demonstrated the broad applicability of DoubletSeeker, including its potential capacity in screening drugs that target membrane protein interactions, such as blocking‐antibody discovery.

The user‐friendly signature of DoubletSeeker makes it easily implementable in a basic biological laboratory. For TCR antigen discovery, DoubletSeeker enables T cell antigen identification with the ability to test a large number of antigens, screen multiple MHC alleles, perform double‐blind screening, and overcome limitations of small antigen/TCR pools during “library‐on‐library” screening. A major limitation to the clinical application of the TCR‐T‐based strategy is the lack of efficient techniques for the discovery of the cognate antigens of a large number of orphan TCRs.^[^ ^28^ ^]^ In recent decades, pMHC tetramers have been extensively used to identify antigen‐specific T cells and facilitate high‐throughput screening by coupling with DNA barcoding. However, the application of pMHC tetramers is constrained by the labor consumption of peptide synthesis, the inconvenient assembly of pMHC tetramers, and the requirement for pre‐designed antigen information. Various cell‐based methods,^[^ ^29^ ^]^ such as T‐Scan, TCR‐MCR, signaling and antigen‐presenting bifunctional receptors (SABRs) and trogocytosis,^[^ ^11^ ^]^ have also been developed to decipher the antigen specificity of specific TCRs. However, these cell‐based methods are still constrained by time‐consuming multi‐round screening, have low sensitivity for low‐affinity antigens, and are unable to be used for “library‐on‐library” screening. DoubletSeeker overcomes these limitations and has the capacity to identify TCR‐antigen interactions across various MHC alleles (MHC I and MHC II) and diverse TCR types, which would significantly enhance our understanding of antigen recognition and immune responses on a broader scale. Moreover, DoubletSeeker supports a wider range of application scenarios compared to pMHC tetramers and cell‐based methods. It offers a bidirectional screening tool, facilitating the identification of both the TCR specific to a given antigen and the cognate antigen recognized by an orphan TCR.

Several “library‐on‐library” TCR antigen screening techniques have been established, such as the yeast display system‐based YAMTAD, and engineered viral‐based ENTER and RAPTR. The application of YAMTAD is hindered by the inherent drawbacks in yeast‐display methods, including low stability of displayed proteins on the yeast surface and the potential for inaccurate antigen presentation. In contrast, engineered viral‐based display systems address the limitations of the yeast display system but face challenges in preparing large‐scale virus‐displaying libraries, and their suitability for displaying protein complexes is uncertain. The mammalian cell display system used in DoubletSeeker offers a convenient approach for generating large‐scale antigen or TCR libraries while maintaining proper folding and modifications of membrane proteins. This endows DoubletSeeker to effectively enrich doublets that accurately represent genuine interactions. Furthermore, DoubletSeeker provides a reliable means of identifying TCR‐antigen interactions through high‐throughput “library‐on‐library” screening, offering distinct advantages in terms of accessibility and screening scale. By expanding the repertoire of TCR‐antigen pairings, including the desired information on MHC II‐restricted TCR/antigen interactions, DoubletSeeker would offer sufficient training data for studying T cell antigen‐specific recognition through in silico methods.^[^ ^28^ ^]^

DoubletSeeker does have certain limitations. First, the time and conditions required for doublets formation may vary depending on the specific receptor‐ligand interactions and the types of presenting cells involved. Thus, exploring and optimizing the detection conditions based on the specific experimental requirements is crucial. Second, the formation of doublets is primarily applicable to protein overexpression systems and may not be as effective for screening primary T cells or endogenously presented antigens. Because tumor cells naturally exhibit low levels of endogenously presented antigens on their surfaces, capturing interactions between these cells and primary T cells, which possess diverse T‐cell receptor (TCR) repertoires, remains challenging for DoubletSeeker. As a result, there is a need for further improvement in the sensitivity of DoubletSeeker for primary cells. Nonetheless, our research suggests that, given its ability to identify OVA‐specific primary OT‐1 T cells, DoubletSeeker could serve as an alternative method for enriching antigen‐specific T cells from peripheral blood.

Overall, DoubletSeeker represents a significant advancement in the field of intracellular communication by enabling the high‐throughput capture of ligand‐receptor interactions. It has the potential to revolutionize the study of immune cell communication and has the potential to greatly enhance our understanding of antigen recognition. DoubletSeeker facilitates comprehensive investigations into the adaptive immune response and as such, opens up avenues for the engineering of such responses against disease.

## 4 Experimental Section

### Cells

HEK293T (ATCC, CRL‐3216), A549 (ATCC, CCL‐185), and SK‐BR‐3 (ATCC, HTB‐30) cells were cultured in Dulbecco's modified Eagle's medium (DMEM) (Gibco) supplemented with 10% (v/v) fetal bovine serum (FBS) (Gibco) and 1% (v/v) penicillin‐streptomycin (P/S) (Gibco). Jurkat E6‐1 (ATCC, TIB‐152), K562 (ATCC, CCL‐243), Raji (ATCC, CCL‐86), and J76 cells were cultured in RPMI 1640 medium (Hyclone) supplemented with 10% (v/v) FBS, 1% (v/v) P/S, 0.1 M HEPES (Gibco), 1 mm sodium pyruvate (Gibco), 1% (v/v) non‐essential amino acids (NEAA) (Gibco) and 50 µm β‐mercaptoethanol (Sigma–Aldrich). Primary human peripheral blood mononuclear cells (PBMCs) from healthy donors were purchased from Sailybio, and cultured in RPMI 1640 medium supplemented with 5% (v/v) human serum (Gemini), 1% (v/v) P/S, 0.1 m HEPES, 1 mm sodium pyruvate, 1% (v/v) NEAA, 1% (v/v) glutaMAX (Gibco), and 50 µm β‐mercaptoethanol in the presence of recombinant human IL‐2 (300 U/ml, Peprotech). All cells were cultured at 37 °C with 5% CO_2_.

### DNA Constructs

The retroviral vector encoding TCR genes and the lentiviral vector encoding SCT genes were constructed as previously described.^[^ ^10^ ^]^ Briefly, either human or murine TCRs were designed using the LNGFR‐P2A‐TCRα‐F2A‐TCRβ format and integrated into the MSGV retroviral vector. Peptide‐MHC SCT composed of antigenic peptide (MART‐1/HLA‐A*02:01, ELAGIGILTV; NY‐ESO‐1/HLA‐A*02:01, SLLMWITQV; NY‐ESO‐1/HLA‐B*18:01, LEFYLAMPF; NY‐ESO‐1/HLA‐C*03:04, FATPMEAEL; USP‐7/HLA‐A*02:01, YLYHRVDVI; MART‐1 variants/HLA‐A*02:01, AAGIGILTV, AAGIGILTVI, EAAGIGILTV, GIGILTVIL, and ILVILGVL), β2‐microglobulin, and HLA domains via flexible glycine‐serine linkers were prepared with a disulfide trap modification expressed in a lentiviral vector encoding an enhanced green fluorescent protein (eGFP). In addition, the MHC‐II restricted antigenic peptide (GAD2_555‐567_, NFFRMVISNPAAT) was linked with HLA‐DRB1 and HLA‐DRA genes via flexible glycine‐serine linkers and then subcloned into a lentiviral vector. The lentiviral or retroviral vectors encoding a blue fluorescent protein (BFP) or eGFP were used in the construction of various membrane proteins, including GPI‐anchored cytokines, CD19‐, HER2‐, EGFR‐CAR, CD8, CD40, CD40L.

### Cell Lines Construction

Retroviruses and lentiviruses were produced in HEK‐293T cells using polyethyleneimine (PEI) (Polysciences) as a transfection reagent, along with packaging plasmid vectors (pRD114 and pHIT60, or psPAX2 and pMD2.G) according to the manufacturer's protocol. After 48 h of transfection, the viruses were filtered through 0.45 µm syringe filters and stored at −80 °C until further use. The cells were spin‐infected with the virus and supplemented with 10 µg mL^−1^ polybrene at 2500 r.p.m. at 30 °C for 90 min. After two days of infection, cells expressing the desired proteins were sorted by FACS.

### Primary T Cell Activation and Retroviral Transduction

Anti‐human CD3 (1 µg mL^−1^, Biolegend) and anti‐human CD28 (1 µg mL^−1^, Biolegend) antibodies were used to activate PBMCs. After 48 h of activation, 1 × 10^6^ PBMCs per 24 well plates were spin‐infected with the virus and fresh medium containing 300 U mL^−1^ IL‐2 and 1 µg mL^−1^ anti‐human CD28 was added. LNGFR or mTCRβ were used to quantify the infection efficiency.

### A2‐Restricted SCT and Neoantigen SCT Library Preparation

The A2‐restricted SCT cDNA library and the neoepitopes SCT cDNA library were generated as previously described.^[^ ^10^ ^]^ Briefly, a pool of oligonucleotides encoding epitopes was synthesized and utilized as the template for PCR amplification. The PCR‐amplified oligonucleotides were then inserted into a BsmBI‐digested lentiviral vector encoding eGFP via flexible glycine‐serine linkers along with β2‐microglobulin and HLA‐A2 structural domains to form SCT by in‐fusion cloning.

### TCR Library Preparation

To introduce random mutations in the CDR3β region of the TRBV gene in M1W‐TCR, 5′‐AATTTCCCCCTGATCCTCGAGTCGCCCAGCCCCAACCAGACCTCTCTGT ACTTCTGTGCCAGCAGTTTANNNNNNNNNNNNNNNNNNCCCCTCCACTTTGGGAACGGGACGCGTCTCACTGTGACAGAG‐3′ was used as the template and then amplified with 5′‐AATTTCCCCCTGATC‐3′ as the forward primer and 5′‐CTCTGTCACAGTGAG‐3′ as the reverse primer. After amplification, the PCR amplicons were digested with XhoI and MluI and then ligated into XhoI and MluI‐digested M1W‐TCR retroviral vector. The product of the ligation reaction was transformed into TG1 electrocompetent cells by the electroporation transformation method. Subsequently, all the transformed bacteria were spread on LB agar plates. The plasmid midiprep kit (CWBIO) was employed to isolate and purify the retroviral plasmid. In the case of M1W‐TCR pool‐J76 cells used in TCR library screening, the retroviral particle production was mixed with 0.1% WT M1W‐TCR virus, and subsequently, CD8‐J76 cells were infected with the virus at an MOI of 4. For M1W‐TCR pool‐J76 cells used in library‐on‐library screening, the CD8‐J76 cells were infected with the virus at an MOI of 1 following retroviral particle production. Then, the cells were sorted to isolate the group that exhibited positive expression of the TCRs. These M1W‐TCR pool‐J76 libraries were then subjected to further screening and analysis.

### Doublet Formation Assay

For doublets formation experiments, cells were labeled with indicated CellTrace dyes at 37 °C for 30 min as the experimental design. After the second washing, co‐incubations of ligand‐expressing cells and receptor‐expressing cells were set up in 1.5 ml tubes at a ratio of 1:5 (30 000–50 000 cells in 75 µL incubation buffer, PBS containing 0.5% BSA and 1 mm EDTA) and co‐incubated for 30 min at 37 °C. Ratios for co‐incubation experiments involving TCR‐pMHC interactions are indicated in the text or figure legends. 500 µL FACS buffer or 4% polyoxymethylene was then added to the co‐incubation. The formation of doublets was then analyzed by flow cytometry. For the screening of A2‐restricted SCT and neoantigen SCT libraries, co‐incubations of SCT library‐expressing cells and TCR‐expressing cells were set up in 1.5 mL tubes at a ratio of 1:1 (300 000 cells in 200 µL incubation buffer). After a 30 min incubation, 800 µL of 4% polyoxymethylene was added to the mixture. For M1W‐TCR pools‐J76 library screening, co‐incubations of MART‐1‐K562 and M1W‐TCR pools‐J76 library were set up in 1.5 mL tubes at a ratio of 1:1 (300 000 cells in 200 µL incubation buffer). After a 30 min incubation, 800 µL of FACS buffer was added to the mixture.

### Visualization of Doublets by Confocal Microscopy

Cells labeled with either CTV or CMFDA were subjected to the doublet formation assay as previously described. The co‐incubated cells were then plated on a confocal dish, and the formation of doublets was detected using a confocal microscope (TCS‐SP6, Leica) with 405 and 488 lasers.

### Flow Cytometry and Sorting

Ligand‐expressing or receptor‐expressing cells were stained with flow cytometry antibodies for 30 min at 4 °C, washed with FACS buffer twice, and then subjected to flow cytometry. The top 10% of cells that exhibited the highest expression of the target proteins were enriched for further analysis. For the cell co‐incubation assay, the signal of different CellTrace dyes was measured by flow cytometry.

### PCR Amplification and Deep Sequencing

Genomic DNA from the sorted SCT‐K562 cells from A2‐restricted SCT and neoantigen SCT library screening was extracted using the Nucleic acid extraction kit (CWBIO), and then used as a template for barcoded PCR amplification, with the following program: 95 °C for 2 min; 35 cycles of 95 °C for 20 s, 63 °C for 10 s, 72 °C for 15 s; final extension of 72 °C for 1 min per kb. The primers used in PCR amplification were the following: SCT‐Forward, 5′‐AATGATACGGCGACCACCGAG ATCTACACTC TTTCCCTACACGACGCTCTTCCGATCTGGCCTGCTTTGTTTGCC‐3′; SCT‐Reverse, 5′‐GTGACTGGAGTTCAGACGTGTGCTCTTCCGATCTCCTCCACCA CCGCTACCTC‐3′; and adaptor‐index reverse primers. The amplified products were purified using DNA, RNA, and protein purification kits (NucleoSpin) according to the manufacturer's protocol, and then were sequenced using an Illumina HiSeq X Ten (Novegene).

### Single Doublets PCR

Single doublets were sorted using FACS into ice‐cold 96‐well plates (Nest), with each well containing 20 µL lysis buffer. The lysis buffer was supplemented with RNase inhibitor (Transgene) at a concentration of 1 U µL^−1^. Immediately after cell sorting, each plate was covered with an adhesive film (Beyotime), briefly spun down in a centrifuge, and then frozen at −80 °C for further analysis and storage. For the pre‐amplification of CDR3β sequences, the primer mix included a 10 µm concentration of primers designed to target the TRBV region (forward primer) and the TRBC region (reverse primer) for endogenous transcripts of the TCRβ chain. Similarly, for the pre‐amplification of antigen sequences, the primer mix consisted of a 10 µm concentration of primers targeting the signal peptide region (forward primer) and the β2M region (reverse primer). To prepare the qRT‐PCR reaction mixture, the primer mixtures were respectively added into the OneStep qRT‐PCR 5 × reaction mix (Qiagen), and DNase/RNase‐free distilled water was added to adjust the final volume to 15 µL^−1^. The plate containing the single doublets in the lysis mix was thawed on ice and briefly spun down in a centrifuge. Then, the contents were aliquoted to 10 µL per reaction and added into the respective qRT‐PCR reaction mix as described. This procedure enabled the amplification of both TCR and antigen sequences for each doublet. The reverse transcription step was performed using a thermal cycler (lid temperature 70 °C) for 30 min at 50 °C. Following this, PCR was performed with the following cycle conditions: 95 °C for 5 min; six cycles of 95 °C for 20 s, 60 °C for 60 s, and 70 °C for 15 s; final extension at 70 °C for 1 min, followed by a 4 °C hold. Nested PCRs for cloning target CDR3β region and antigen sequence were performed using the 2× PrimeSTAR (Takara) and nest‐designed primers. Cycling conditions were as follows: 95 °C for 2 min; 35 cycles of 95 °C for 20 s, 60 °C for 20 s, 72 °C for 15 s; final extension of 72 °C for 1 min.

### Peptide Loading in Antigen‐Presenting Cells

Lyophilized peptides (LifeTein) were redissolved in dimethylsulfoxide (Sigma‐Aldrich) at a concentration of 10 mm. The peptides were then further diluted in water to achieve a final concentration of 100 nm. A2‐K562‐luciferase cells (50 000 total, 0.5 × 10^6^ cells mL^−1^) were pulsed with peptide dilution in a 96‐well U‐bottom plate and incubated for 2 h at 37 °C. After the incubation, 100 µL of the medium was added to each well, and the plate was centrifuged for 5 min at 1500 r.p.m. The cells were subsequently washed once with 200 µL of medium and resuspended in 100 µL of medium in preparation for the cytotoxicity assay.

### Co‐Culture of Primary Human T Cells and Cytotoxicity Assay

Primary T cells expressing the TCR of interest and A2‐K562‐luciferase cells pulsed with the corresponding peptide were co‐cultured in the wells of a 96‐well U‐bottom plate. The ratio of effector cells to target cells was maintained at 5:1. After 6 h of co‐culture, the plate was centrifuged for 5 min at 1500 r.p.m. The cells were then resuspended in 50 µL of PBS and frozen at −20 °C for 1 h to facilitate cell lysis. To assess T‐cell mediated cytotoxicity, the intercellular luciferase activity of the surviving target cells was measured. Briefly, the luciferase substrate was added to each well at a 1:1 ratio. The plate was gently agitated in the dark to stabilize the luminescent signal. The luminescence of each well was then measured using a Multifunctional microporous plate reader (SpectraMax). The average values from triplicate wells were calculated, and the percent lysis was determined using the following equation: % specific lysis = 100 × (maximum fluorescence value – test fluorescence value) / maximum fluorescence value.

### Statistical Analysis

Statistical analysis was performed using GraphPad Prism version 9.0. An unpaired t‐test was used to assess the statistical significance. Data are represented as mean ± SEM in bar blots. P values are as indicated in the figure legends.

## Conflict of Interest

The authors declare no conflict of interest.

## Author Contributions

Y.W., Z.W., and J.Y. contributed equally to this work. G.L. conceived the idea. G.L., Z.W., and Y.W. designed the overall experiments, analyzed all the data, and wrote the manuscript. Y.W. and J.Y. performed the experiments. Y.L. analyzed the sequencing data. X.L., L.F., J.W., and G.L. reviewed the article. J.W. and G.L. supervised this research.

## Supporting information

Supporting Information

## Data Availability

The data that support the findings of this study are available from the corresponding author upon reasonable request.
