# Supplementary material for: Deciphering Membrane‐Protein Interactions and High‐Throughput Antigen Identification with Cell Doublets
Source: Adv Sci (Weinh). 2024 Feb 11;11(13):2305750. doi: 10.1002/advs.202305750 (PMC10987144; doi:10.1002/advs.202305750)
Supplement: Supplementary file 1 — Supporting Information [file ADVS-11-2305750-s001.pdf]

ADVANCED SCIENCE
Open Access

Supporting Information

for *Adv. Sci.*, DOI 10.1002/advs.202305750

Deciphering Membrane-Protein Interactions and High-Throughput Antigen Identification with Cell Doublets

Yuqian Wang, Zhe Wang, Juan Yang, Xiaobo Lei, Yisu Liu, Luke Frankiw, Jianwei Wang and Guideng Li**

Supplementary Figures for

Deciphering membrane-protein interactions and high-throughput antigen identification with cell doublets

Yuqian Wang[1,2†], Zhe Wang[1,2†], Juan Yang[1,2†], Xiaobo Lei[4], Yisu Liu[1,2], Luke Frankiw[3], Jianwei Wang[4*], and Guideng Li[1,2*]

[1]National Key Laboratory of Immunity and Inflammation, Suzhou Institute of Systems Medicine, Chinese Academy of Medical Sciences & Peking Union Medical College, Suzhou 215123, Jiangsu, China.

[2]Key Laboratory of Synthetic Biology Regulatory Element, Suzhou Institute of Systems Medicine, Chinese Academy of Medical Sciences & Peking Union Medical College, Suzhou 215123, Jiangsu, China.

[3]Department of Pediatrics, Boston Children's Hospital, Boston, MA, 02115, US

[4]NHC Key Laboratory of Systems Biology of Pathogens, Institute of Pathogen Biology, Chinese Academy of Medical Sciences and Peking Union Medical College, 100730, Beijing, China.

[†]These authors contributed equally to this work.

***Correspondence:**

lgd@ism.cams.cn (G.L.)

wangjw28@163.com (J.W.)

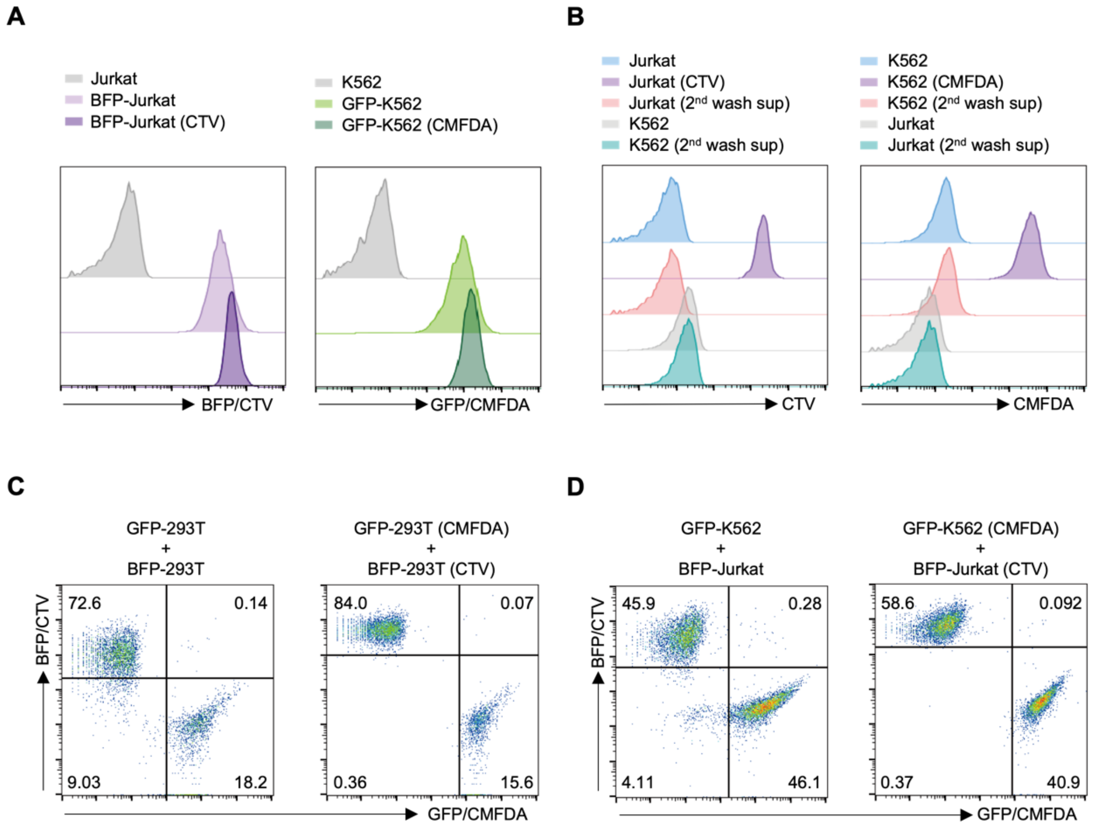

Supplementary Figure 1. Optimization of cell tracing method for minimizing signal-to-noise ratio of DoubletSeeker. (**A**) Representative image shows the background fluorescence in Jurkat, BFP-Jurkat and CTV-labeled BFP-Jurkat cells after 405 nm excitation, and in K562, GFP-K562 and CMFDA-labeled GFP-K562 cells after 488 nm excitation. (**B**) Fluorescence histogram of CTV and CMFDA in Jurkat and K562 cells, with or without staining with the secondary washing supernatant (2nd wash sup) from cells following the indicated fluorescent dye staining. CTV-labeled Jurkat cells and CMFDA-labeled K562 cells served as positive controls for CTV and CMFDA fluorescence, respectively. (**C** and **D**) Two-color contour plots showing the non-specific cell doublets in indicated co-incubation groups. CTV: CellTrace^{TM} Violet, CMFDA: CellTrace^{TM} CMFDA, BFP-Jurkat (CTV): CTV-labeled BFP-Jurkat cells, GFP-K562 (CMFDA): CMFDA-labeled GFP-K562 cells, Jurkat (CTV): CTV-labeled Jurkat cells, K562 (CMFDA): CMFDA-labeled K562 cells, GFP-293T (CMFDA): CMFDA-labeled GFP-293T cells, BFP-293T (CTV): CTV-labeled BFP-293T cells. Data are representative of three independent experiments.

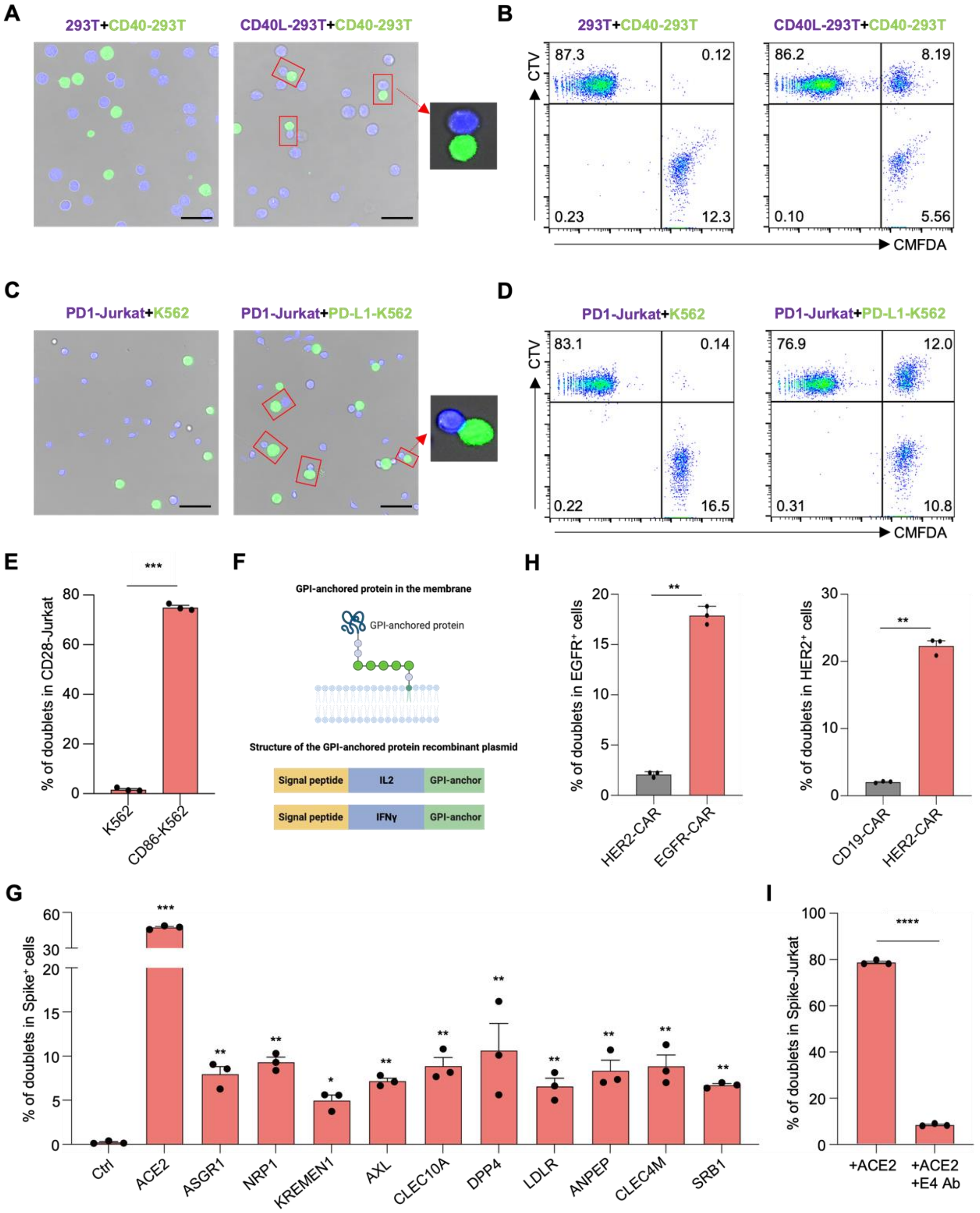

Supplementary Figure 2. DoubletSeeker's ability to decipher specific interactions between ligands and receptors for the indicated molecules. (**A** to **D**) Representative of doublets in CD40-293T (CTV-labeled, blue) co-incubated with control 293T or CD40L-293T cells (CMFDA-labeled, green) (A, B), as well as PD1-Jurkat cells (CTV-labeled, blue) co-incubated with control K562 cells or PD-L1-K562 cells (CMFDA-labeled, green) (C, D), analyzed by confocal microscopy (A, C) and flow cytometry (B, D). Red rectangle boxes mark the presence of doublets. Scale bars, 50 μm. (**E**) Formation of doublets was tested in CD28-expressing-Jurkat cells after co-incubation with either control K562 cells or CD86-expressing-K562 cells. (**F**) Schematic illustration depicts the GPI-anchored protein attached to the cell membrane and

provides an overview of the plasmid structure. (**G**) Formation of doublets after co-incubation of the cells expression Spike protein of SARS-CoV-2 (CTV-labeled) with cells expression different known host cell receptors (CMFDA-labeled). (**H**) Formation of doublets was tested between EGFR-CAR/EGFR and HER2-CAR/HER2. (**I**) Bar plots showing the percentage of doublets among Jurkat-Spike cells co-cultured with K562-ACE2 cells in the presence of SARS-CoV-2 neutralizing antibody E4 (E4 Ab). CTV: CellTrace^{TM} Violet, CMFDA: CellTrace^{TM} CMFDA. Data are represented as the means ± SEM. n=3. *P < 0.05, ***P* < 0.01, ****P* < 0.001, and *****P* < 0.0001(unpaired two-tailed Student's t-test). Data are representative of three independent experiments (A to D).

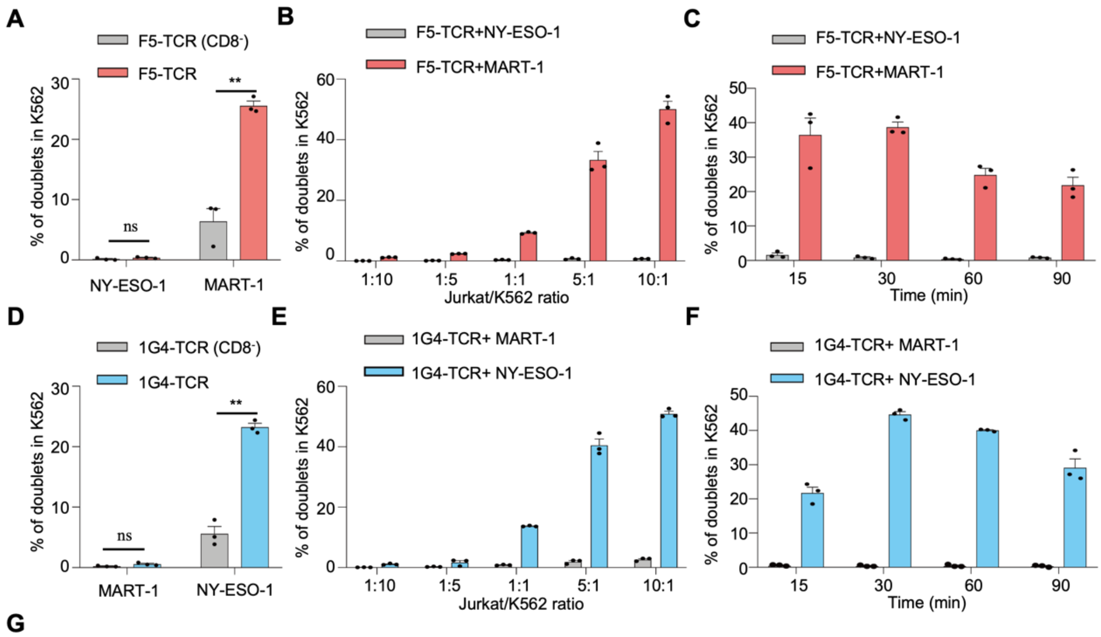

G

Ligand	Receptor	Affinity (nM)	% of doublets	References
CD19	CD19-CAR	0.17	54.77	EBioMedicine. 2020;58:102931
Spike	E4 antibody	0.82	67.17	Cell Biosci. 2022;12(1):63
IL-2	IL-2R	10.00	32.40	Math Med Biol. 2018;35(1):79-119
IFNγ	IFNγR	10.00	5.28	J Interferon Cytokine Res. 2014;34(1):41-51
Spike	ACE2	12.40	47.47	Cell Res. 2022;32(1):24-37
Spike	CLEC4M	16.00	8.87	Cell Res. 2022;32(1):24-37
Spike	KREMEN1	19.30	4.97	Cell Res. 2022;32(1):24-37
CD40L	CD40	20.00	60.27	Arthritis Res Ther. 2021;23(1):5
EGFR	EGFR-CAR	40.00	17.90	MAbs. 2020;12(1):1688616
Spike	ASGR1	94.80	7.96	Cell Res. 2022;32(1):24-37
Spike	NRP1	166.20	9.32	Pain. 2021;162(1):243-252
Spike	AXL	882.00	7.18	Sci Adv. 2023;9(2):eade0869
CD28	CD80	4000.00	31.63	Front Immunol. 2016; 7: 24
NY-ESO$_{SLLMWITQV}$-A2	1G4-TCR	4300.00	45.53	Biotechniques. 2017;62(3):123-130
MART-1$_{ELAGIGILTV}$-A2	F5-TCR	5400.00	34.77	Biotechniques. 2017;62(3):123-130
PD-L1	PD-1	8200.00	51.30	Proc Natl Acad Sci. 2015;112(47):E6506-14
MART-1$_{ELAGIGILTV}$-A2	M1W-TCR	15000.00	23.30	Biotechniques. 2017;62(3):123-130
CD28	CD86	20000.00	75.10	Front Immunol. 2016; 7: 24
Spike	CLEC10A	32000.00	8.88	Chem Soc Rev. 2023; 52(11): 3663–3740
MART-1$_{EAAGIGILTV}$-A2	F5-TCR	37000.00	15.97	Mol Ther. 2019;27(2):300-313

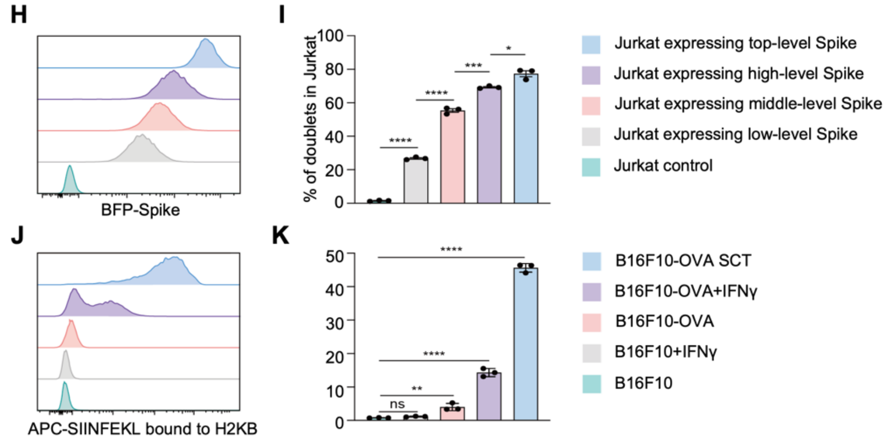

Supplementary Figure 3. Exploration of optimal experimental conditions for DoubletSeeker in the identification of TCR-pMHC interactions. (**A** and **B**) Comparison of doublets formation in MART-1-K562 or NY-ESO-1-K562 cells after co-incubation with CD8^{+} or CD8^{-} (CD8 non-expressed) F5-TCR-Jurkat, or 1G4-TCR-Jurkat cells (TCR-expressing cells: SCT-expressing cells=1:1). (**C** to **F**) Comparison of the percentage of doublets in MART-1-K562 or NY-ESO-1-K562 cells after co-

incubation with Jurkat cells expressing cognate or noncognate TCRs at different ratios (TCR-expressing cells: SCT-expressing cells=1:10, 1:5, 1:1, 5:1, 10:1) (C, D) or at different times (15, 30, 60, 90 min) (E, F). (**G**) List of affinities between ligands and corresponding receptors. (**H**) Representative histogram showing Jurkat-Spike cells with varying levels of Spike expression. (**I**) Bar plots showing the percentage of doublets among Jurkat-Spike cells from (H) after co-culturing with K562-ACE2 cells. (**J**) Flow cytometric analysis of SIINFEL-H2KB expression on B16F10 cells and B16F10-OVA cells with or without IFNγ pre-treatment. B16F10-OVA: B16F10 cells express OVA protein, B16F10-OVA SCT: B16F10 cells express OVA-derived SIINFEKL epitope-H2KB SCT. (**K**) Percentage of doublets among various B16F10 cells from (J) after co-culture with primary OT1 T cells. Data are represented as the means ± SEM. *n*=3. ***P* < 0.01 and ns indicates no significance (unpaired two-tailed Student's t-test).

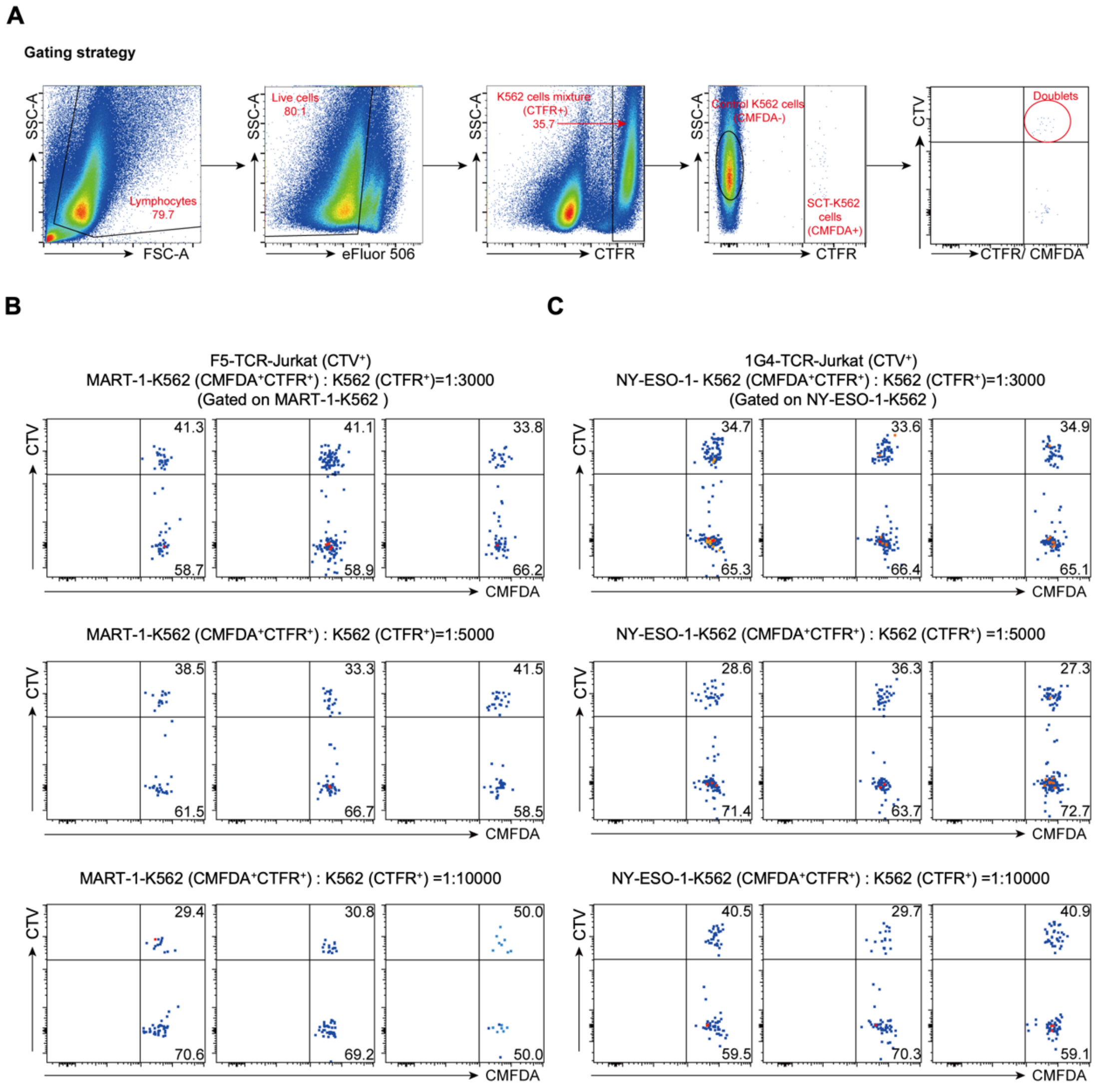

Supplementary Figure 4. Flow cytometry plots for proof-of-concept screening. (**A**) Flow cytometry gating strategy for proof-of-concept screening. (**B**) Flow cytometry plots for doublets identification in a mixture containing cognate MART-1-K562 (B) or NY-ESO-1-K562 (C) cells with noncognate K562 cells at different ratios (1:3000,

1:5000, 1:10000), co-incubated with either F5-TCR-Jurkat cells (B) or 1G4-TCR-Jurkat cells (C). MART-1-K562 and NY-ESO-1-K562 cells were labeled with CMFDA and CTFR, whereas K562 cells were labeled with CTFR. TCR-Jurkat cells were labeled with CTV. CTV: CellTrace^{TM} Violet, CMFDA: CellTrace^{TM} CMFDA, CTFR: CellTrace^{TM} Far-red. *n*=3.

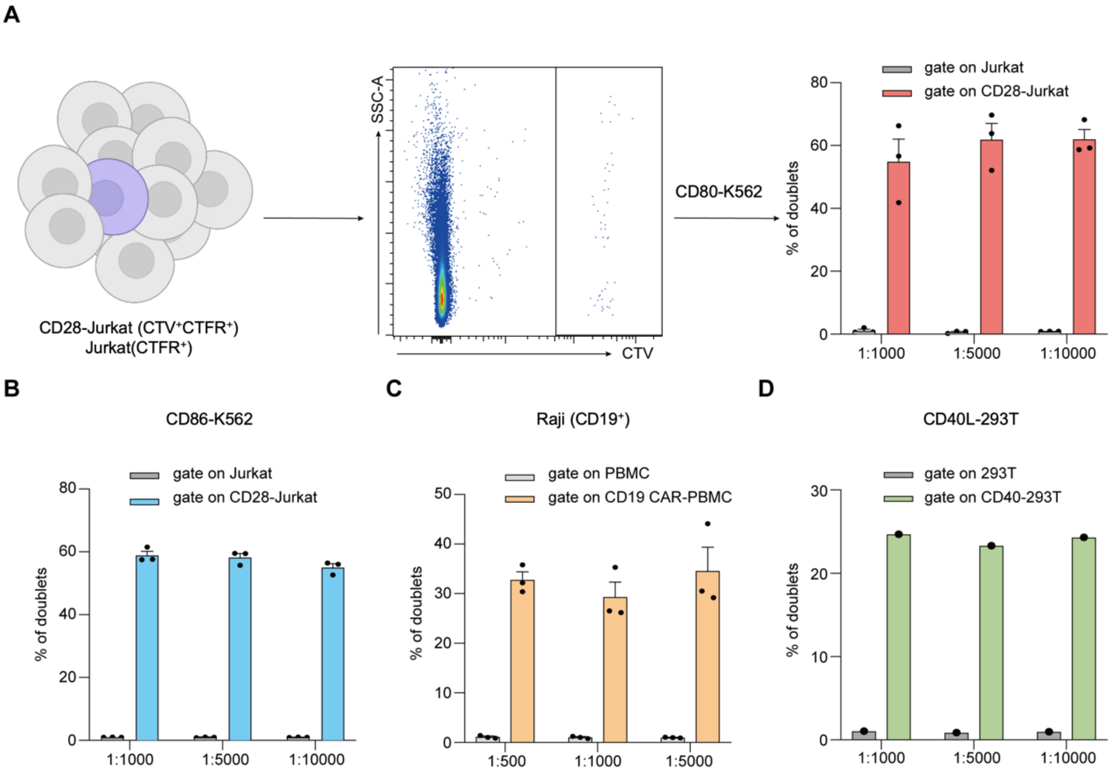

Supplementary Figure 5. Specificity and sensitivity of DoubletSeeker in capturing specific ligand-receptor interactions. (**A**) Schematic and representative Flow cytometry plots for doublets identification in a mixture containing CD28-Jurkat cells with control Jurkat cells at different ratios, co-incubated with CD80-K562 cells. The percentage quantification of doublets in each Jurkat cells are presented on the right. (**B** to **D**) Quantification of the percentage of doublets in each cell population from a mixture containing cells expressing the target protein (CD28, CD19-CAR, and CD40) with control cells at different ratios, following co-incubation with cells expressing the specific ligand (CD86, CD19 and CD40L). Data are represented as the means ± SEM. *n*=1 (D) or 3 (A to C). Data in are representative of three independent experiments (A).

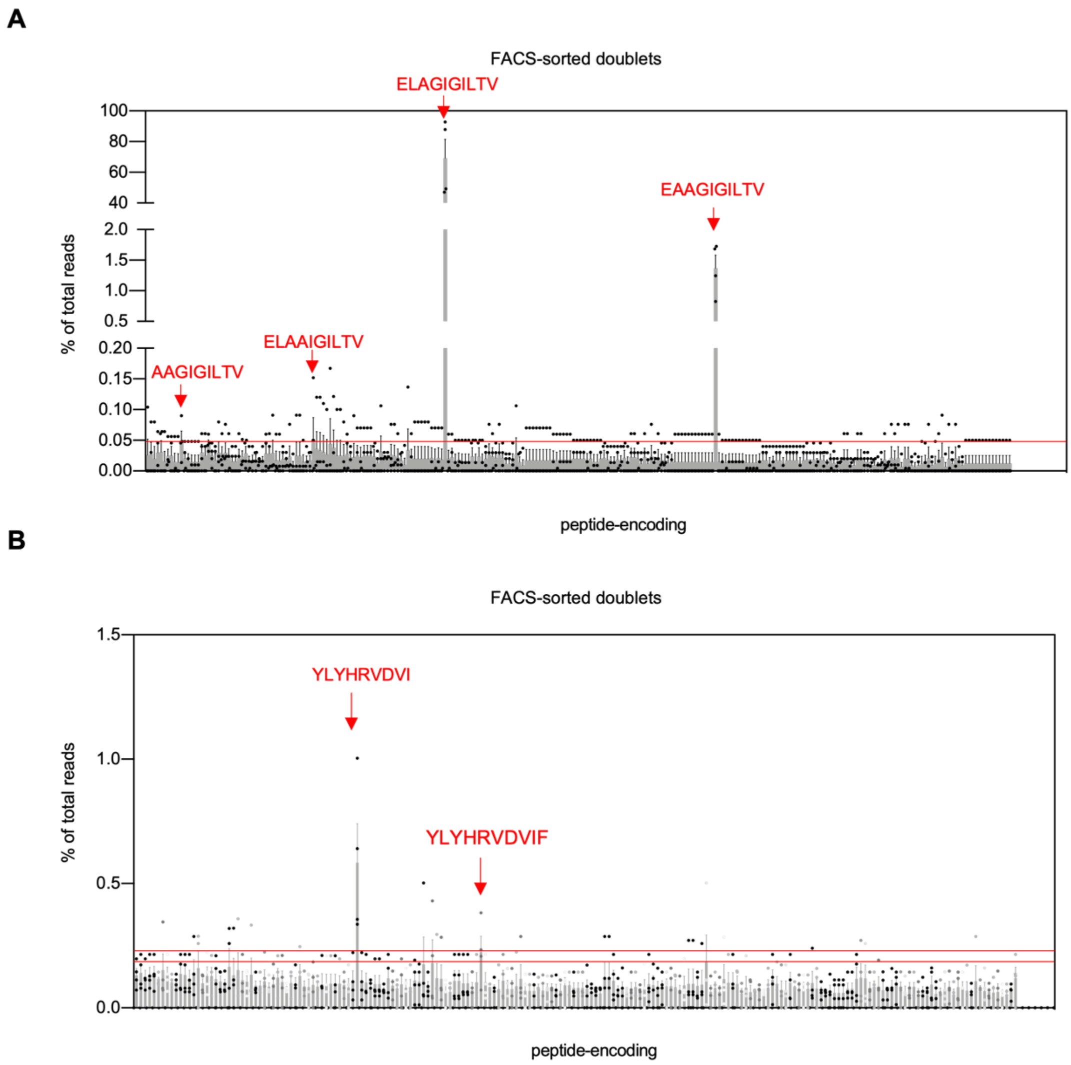

Supplementary Figure 6. DoubletSeeker facilitates the screening of antigens for TCRs. (**A**) Reads of peptides in sorted doublets, obtained from the screening of F5-TCR targeted antigens in the A2-restricted SCT library. (**B**) Reads of peptides in sorted doublets obtained from the screening of Neo-TCR targeted antigens in the neoepitope SCT library. Data are represented as the means ± SEM. *n*=4.

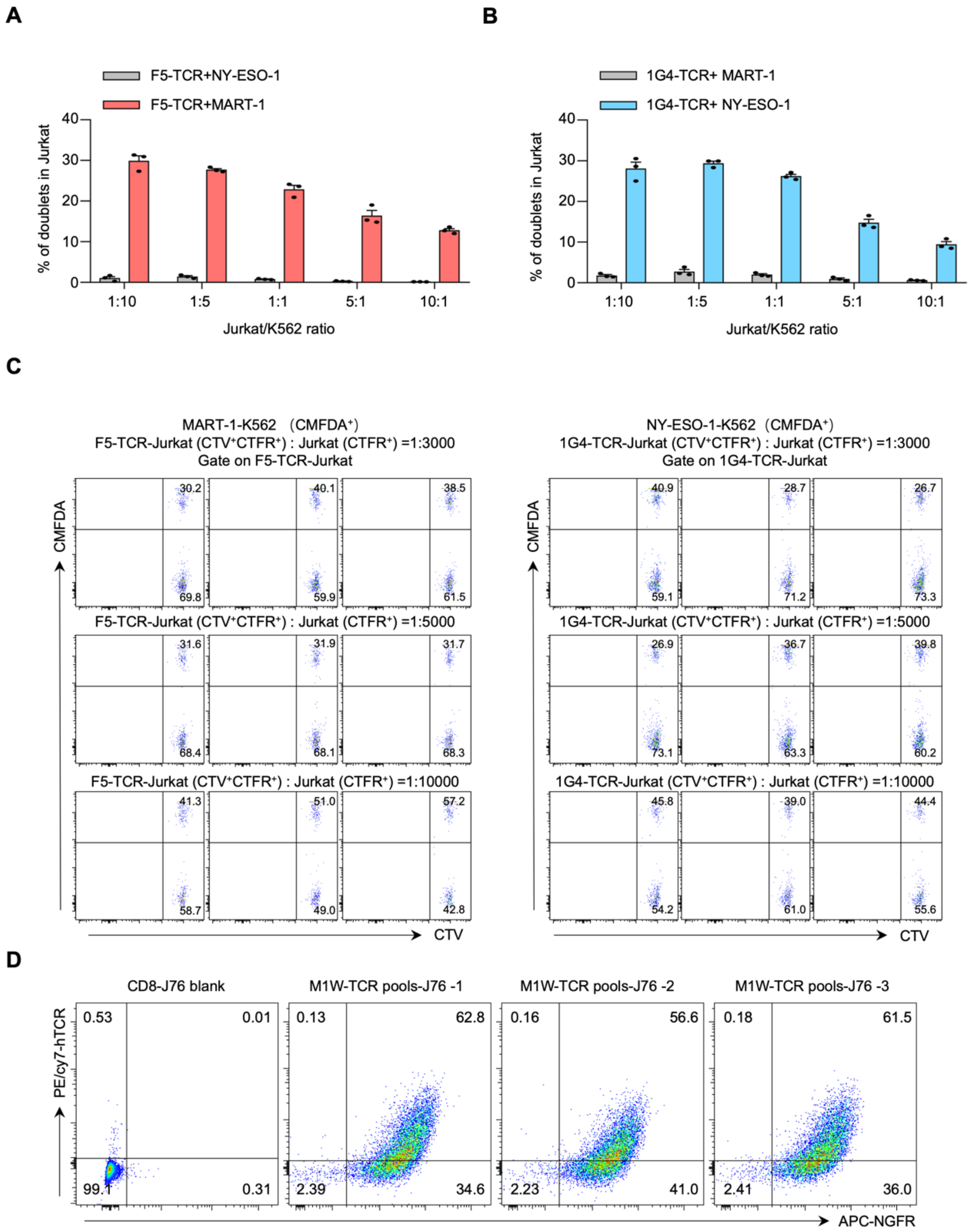

Supplementary Figure 7. Identification of TCRs specific to a given antigen by DoubletSeeker, and the construction of the M1W-TCR pools-J76 library. (**A** and **B**) Comparison of the percentage of doublets in F5-TCR-Jurkat (A) or 1G4-TCR-Jurkat (B) cells after co-incubation with either K562 cells expressing cognate or noncognate antigens at different ratios (1:10, 1:5, 1:1, 5:1 and 10:1). (**C**) Flow cytometry plots illustrating doublets in cognate F5-TCR-Jurkat cells mixed with varying proportions of the noncognate Jurkat cells mixture (left) or in cognate 1G4-TCR-Jurkat cells mixed with different proportions of the noncognate Jurkat cells mixture (right), co-incubated with MART-1-K562 cells (left) or NY-ESO-1-K562 cells (right). Doublets are analyzed by gating on cognate TCR-Jurkat populations (CTV$^+$CTFR$^+$). The colors are assigned

as TCR-Jurkat cells (CTV$^+$CTFR$^+$), noncognate Jurkat cells (CTFR$^+$), SCT-K562 cells (CMFDA$^+$) and specific doublets (CTV$^+$CMFDA$^+$). (**D**) Flow cytometry plots showing the expression of TCR and the co-expressed NGFR in CD8-J76 cells transduced with the M1W-TCR pools at an MOI of 4. CTV: CellTrace^{TM} Violet, CMFDA: CellTrace^{TM} CMFDA, CTFR: CellTrace^{TM} Far-red. Data are represented as the means ± SEM. *n*= 3.

A

	Rank	Read	Percentage
CASSLVGTAGSPLHF (WT)	1	745	9.5%
CASSLGGLWCSPLHF	2	31	0.37%
CASSLVLSRRPPLHF	3	17	0.21%
CASSLGGSAPGPLHF	4	16	0.18%
CASSLATLSSLPLHF	5	15	0.17%
CASSLGIGIGRPLHF	6	11	0.14%
CASSLGICAGLPLHF	7	11	0.14%
CASSLGALAAGPLHF	8	9	0.12%
CASSLGASSRAPLHF	9	9	0.12%
CASSLAGYAQPPLHF	10	8	0.10%

B

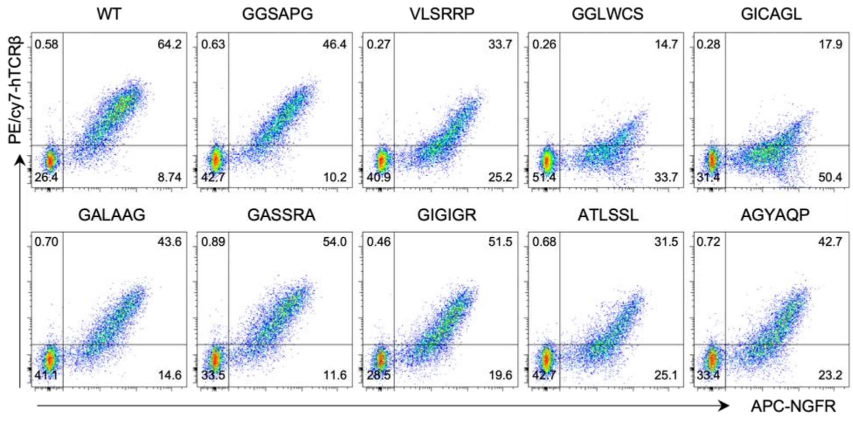

C

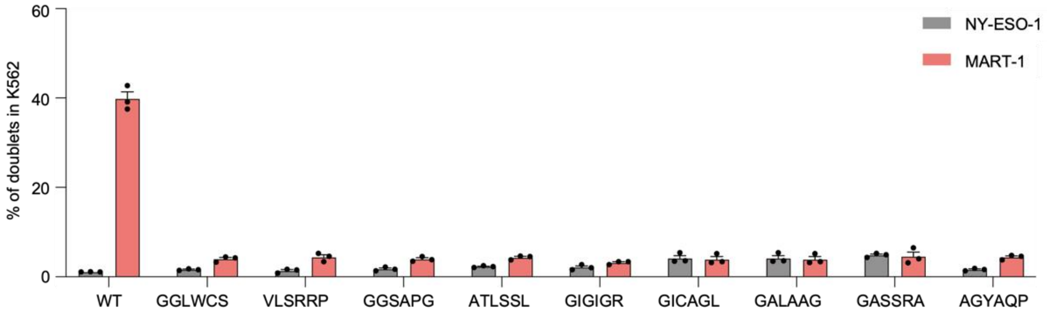

Supplementary Figure 8. Validation of the specificity of enriched M1Wmut TCRs for MART-1 ELAGIGILTV epitope via DoubletSeeker. (**A**) Read counts of the top 10 enriched TCRs in doublets sorted from the co-culture of M1W-TCR pools-J76 library with MART-1-K562 cells. (**B**) Flow cytometry plots showing the expression of TCRs and the co-expressed NGFR in CD8-J76 cells transduced with the WT M1W-TCR, as well as the enriched M1W mutant TCRs ranking from the top 2 to 9. WT M1W-TCR as a positive control. (**C**) Identification of doublets formed in MART-1-K562 or NY-ESO-1-K562 cells following co-culture with CD8-J76 cells from (B). Data are represented as the means ± SEM. *n*= 3. Data are representative of three independent experiments (B).

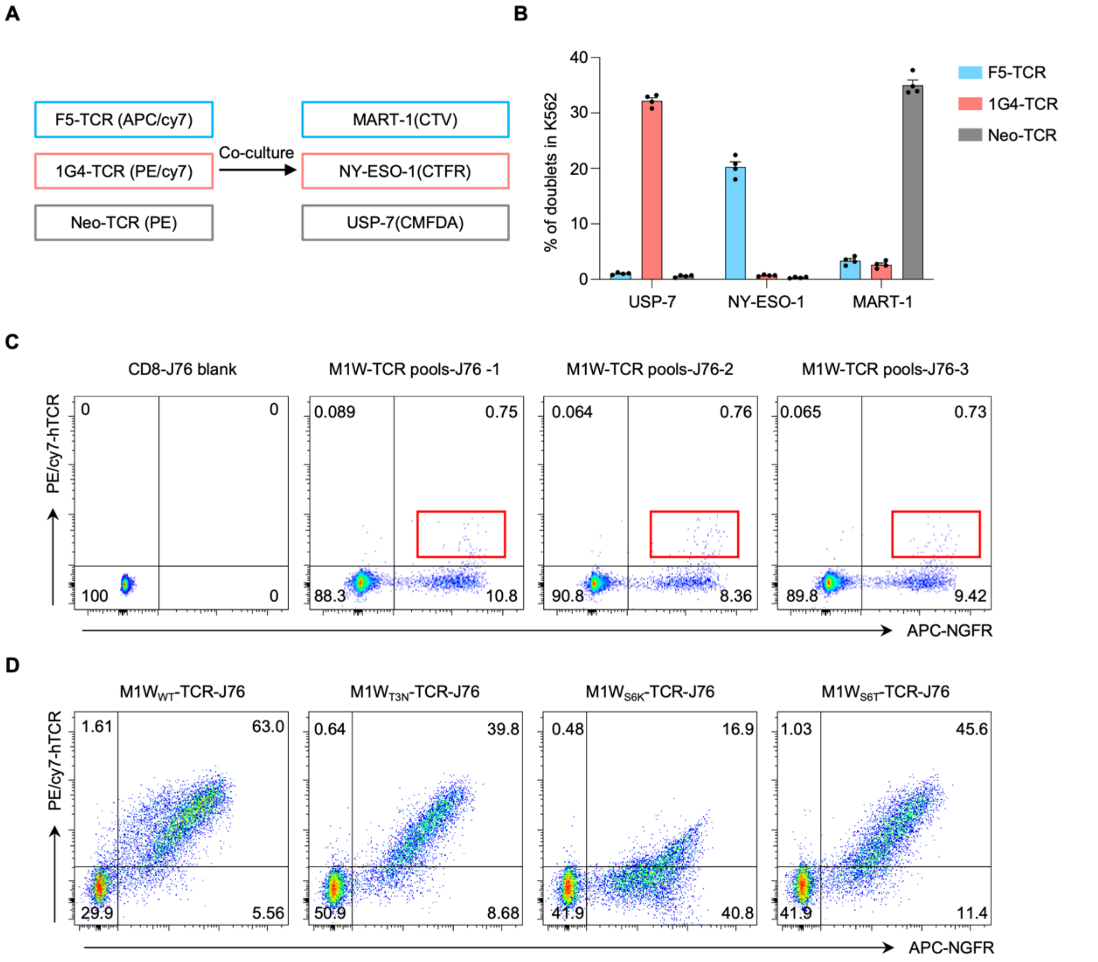

Supplementary Figure 9. The proof-of-concept library-on-library screening strategy and the construction of M1W-TCR pools-J76 library at an MOI of 1.

(**A**) Schematic diagram of the proof-of-concept library-on-library screening. (**B**) Antigen-specific doublets formation in the mixture of F5-TCR-Jurkat, 1G4-TCR-Jurkat, and Neo-TCR-Jurkat cells incubated with MART-1-K562, NY-ESO-1-K562 and USP7-K562 cells mixtures. (**C**) Flow cytometry plots showing the expression of TCR and the co-expressed NGFR in CD8-J76 cells transduced with the M1W-TCR pools at an MOI of 1. (**D**) Flow cytometric assessment of mutant M1W-TCRs, enriched from the "library-on-library" screening presented in Fig6.D, expression in J76 cells. CTV: CellTrace™ Violet, CMFDA: CellTrace™ CMFDA, CTFR: CellTrace™ Far-red. Data are represented as the means ± SEM. *n*= 3.
